# IRE1α Activation in Bone Marrow-Derived Dendritic Cells Modulates Innate Recognition of Melanoma Cells and Favors CD8^+^ T Cell Priming

**DOI:** 10.3389/fimmu.2018.03050

**Published:** 2019-01-04

**Authors:** Bernardita Medel, Cristobal Costoya, Dominique Fernandez, Cristian Pereda, Alvaro Lladser, Daniela Sauma, Rodrigo Pacheco, Takao Iwawaki, Flavio Salazar-Onfray, Fabiola Osorio

**Affiliations:** ^1^Program of Immunology, Laboratory of Immunology and Cellular Stress, Faculty of Medicine, Institute of Biomedical Sciences, Universidad de Chile, Santiago, Chile; ^2^Programa de Doctorado en Biotecnología, Facultad de Ciencias de la Vida, Universidad Andres Bello, Santiago, Chile; ^3^Millennium Institute on Immunology and Immunotherapy, Universidad de Chile, Santiago, Chile; ^4^Program of Immunology, Laboratory of Antitumor Immunity, Faculty of Medicine, Institute of Biomedical Sciences, University of Chile, Santiago, Chile; ^5^Laboratory of Immunoncology, Fundacion Ciencia & Vida, Santiago, Chile; ^6^Departamento de Biologia, Facultad de Ciencias, Universidad de Chile, Santiago, Chile; ^7^Departamento de Ciencias Biológicas, Facultad de Ciencias de la Vida, Universidad Andres Bello, Santiago, Chile; ^8^Laboratorio de Neuroinmunología, Fundación Ciencia & Vida, Santiago, Chile; ^9^Division of Cell Medicine, Department of Life Science, Medical Research Institute, Kanazawa Medical University, Ishikawa, Japan

**Keywords:** IRE1α, XBP1s, UPR, dendritic cell, melanoma, cross-presentation

## Abstract

The IRE1α/XBP1s signaling pathway is an arm of the unfolded protein response (UPR) that safeguards the fidelity of the cellular proteome during endoplasmic reticulum (ER) stress, and that has also emerged as a key regulator of dendritic cell (DC) homeostasis. However, in the context of DC activation, the regulation of the IRE1α/XBP1s axis is not fully understood. In this work, we report that cell lysates generated from melanoma cell lines markedly induce XBP1s and certain members of the UPR such as the chaperone BiP in bone marrow derived DCs (BMDCs). Activation of IRE1α endonuclease upon innate recognition of melanoma cell lysates was required for amplification of proinflammatory cytokine production and was necessary for efficient cross-presentation of melanoma-associated antigens without modulating the MHC-II antigen presentation machinery. Altogether, this work provides evidence indicating that *ex-vivo* activation of the IRE1α/XBP1 pathway in BMDCs enhances CD8^+^ T cell specific responses against tumor antigens.

## Introduction

Dendritic cells (DCs) are an heterogeneous family of leukocytes competent to instruct antigen-specific immune responses (1). Based on surface markers, location, ontogeny, and function, these cells can be divided into plasmacytoid DCs (pDCs) and conventional DCs (cDCs), which are sub-classified into cDC1 and cDC2 subtypes (2). cDC1s express the surface markers XCR1, DNGR-1, and CD103 in non-lymphoid organs, and require the transcription factors Batf3 and Irf8 for development (3–6). On a functional level, cDC1s are highly efficient at priming CD8^+^ T cell responses *in vivo* to cell-associated antigens through a process termed “cross-presentation” (7). On the other hand, cDC2s express the surface markers CD11b and CD172a (SIRPα), the transcription factors Irf4, Klf4, and Notch2 are recognized for modulating CD4^+^ T cell responses (2, 4, 5). In inflammatory settings, blood monocytes can also differentiate into antigen presenting cells that resemble CD11b^+^ DCs and that have been referred to as monocyte-derived DCs (8). Cell equivalents of cDCs/pDCs and monocyte-derived DCs can be generated upon *ex-vivo* treatment with FMS-like tyrosinase kinase 3 ligand (FLT3L) or granulocyte-macrophage colony-stimulating factor (GM-CSF), respectively (9, 10). Remarkably, the process of antigen cross-presentation, which is essential for eliciting cytotoxic T cell immunity against tumors, can be efficiently executed by cDC1s, but also by GM-CSF derived DCs through different transcriptional programs (11).

The remarkable ability to evoke T cell immunity have turned DCs into prominent candidates in the generation of cell-based vaccines, particularly in the field of cancer immunotherapy (12). In light of these findings, the intracellular mechanisms governing the immunogenic function of DCs, and in particular those safeguarding cellular function and homeostasis, are matter of extensive research in cancer immunology.

Although it is well-described that microbes and danger signals are potent elicitors of DC activation, emerging evidence indicates that DCs are also sensitive to a broad variety of stress signals for fine-tuning an activated profile (13). A relevant cellular stress-sensing pathway in DC biology is the unfolded protein response (UPR), which is the adaptive cellular mechanism responsible to maintain the fidelity of the cellular proteome (14). The UPR is triggered by accumulation of misfolded proteins in the ER and it is controlled by three ER-resident signal transducers: inositol requiring enzyme 1 (IRE1) alpha and beta, protein kinase R-like ER kinase (PERK) and activating transcription factor 6 (ATF6) alpha and beta (14, 15). The UPR sensors control the expression of genes involved in the recovery of ER homeostasis and also coordinate the execution of cell death under conditions of irrevocable ER stress (14, 16, 17). The IRE1α arm of the UPR is highly conserved among species and it is the most characterized branch in immunity (18). IRE1α is an enzyme containing a serine/threonine kinase domain and an endonuclease domain. In response to the accumulation of misfolded proteins in the ER, IRE1α dimerize, and trans-autophosphorylate activating its endonuclease domain, which performs an unconventional splicing reaction of the *Xbp1* (X-box binding protein) mRNA, generating the transcription factor XBP1 spliced (XBP1s), a major regulator of ER biogenesis (16). In addition, under certain conditions of chronic ER stress or functional loss of XBP1, IRE1α endonuclease initiates the cleavage of additional mRNAs of diverse nature, in a process named “Regulated IRE1 Dependent Decay” or RIDD (19). RIDD was originally proposed to reduce the ER folding load by alleviating the detrimental effects of ER stress.

The dual function of IRE1α endonuclease has emerged as a relevant regulator of DC homeostasis and function. On one hand, XBP1s is constitutively expressed by DC subsets and high expression of XBP1s is a hallmark of cDC1s (20–22). In addition, cDC1s are highly sensitive to changes in IRE1α signaling; as it is reported that RIDD regulates cDC1 survival in mucosal tissues and curtails their ability to cross-present dead cell-associated antigens (21, 22). Whereas, these studies have uncovered a crucial role for the IRE1α/XBP1s axis in non-activated DCs, it remains to be addressed the contribution of the pathway in the functionality of the different DC lineages upon inflammation. This is a relevant aspect considering that innate recognition is a well-described inducer of DC activation (23) and because several pattern recognition receptors (PRRs) induce IRE1α activation for amplification of proinflammatory cytokines (24–28). Interestingly, in the field of tumor therapy, the role of the IRE1α/XBP1s axis in DCs has shown distinct effects depending on whether the pathway is targeted *ex-vivo* or during the course of tumor growth. On one hand, in models of ovarian cancer it has been reported that XBP1s signaling in tumor-infiltrating DCs curtails their ability to trigger anti-tumor T cell immunity, which in turn promotes tumor growth (29). However, enforced expression of XBP1s in *ex-vivo* generated DCs has shown opposite effects, as it potentiates the efficacy of DC-based vaccines in prophylactic and therapeutic settings (30, 31). Thus, the relevance of IRE1α/XBP1s signaling in DCs has not been fully elucidated and it appears to be dependent on the type of DC targeted, on the experimental setting (*in vivo* or *ex-vivo*) and inflammatory context.

In this study, we report that lysates derived from melanoma cell lines are efficient elicitors of the IRE1α-dependent XBP1s branch of the UPR in bone marrow derived DCs (BMDCs), which favors cross-presentation of a melanoma-associated antigen. Pharmacological blockade of IRE1α endonuclease in BMDCs stimulated with melanoma cell lysates impairs cross-presentation of antigens, without interfering with the MHC-II pathway. Furthermore, BMDCs expressing a mutant isoform of IRE1α that lacks the endonuclease domain were less efficient at inducing CD8^+^ T cell proliferation to a melanoma-associated antigen *in vivo*. Our data indicates that activation of the IRE1α/XBP1s axis in BMDCs *ex-vivo* is required to endure CD8^+^ T cell priming to melanoma antigens. Knowledge derived from this study may be considered in the design of DC-based vaccines for cancer immunotherapy.

## Results

### Innate Recognition of Melanoma Cell Lysates Elicits Activation of IRE1α Endonuclease and the Splicing of *Xbp1* mRNA in BMDCs

**Figure 1:**
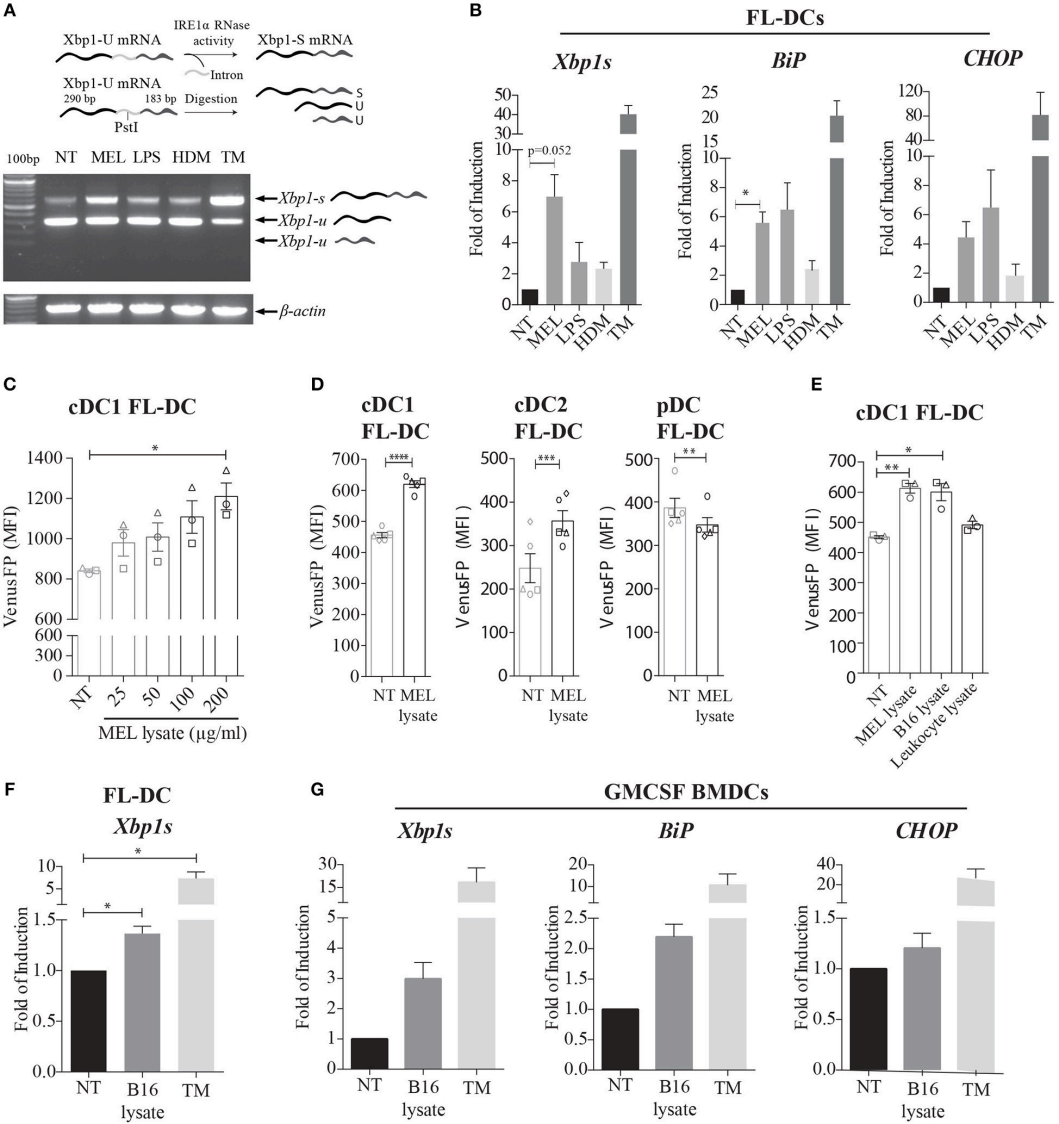
Human and murine melanoma cell lysates induce expression of XBP1s and additional members of the UPR in murine BMDCs. **(A)** FL-DCs were left untreated (NT) or stimulated with 100 μg/ml cell lysate from human melanoma cell lines (MEL), 100 ng/ml lipopolysaccharide (LPS), 50 μg/ml house dust mite extract (HDM), or 1 μg/ml tunicamycin (TM) for 8 h. Expression of *Xbp1s* was determined by a RT-PCR protocol for *Xbp1s* and *Xbp1u* that includes a digestion step with the restriction enzyme PstI. The Pst I digestion site in the intron of *Xbp1u* mRNA allows the distinction between *Xbp1s* and two fragments of *Xbp1u* mRNA. A representative scheme is illustrated. Data is representative of three independent experiments. **(B)** FL-DCs were stimulated as in **(A)** and expression of *Xbp1s, BiP*, and *CHOP* mRNA was measured by qPCR relative to *L27* expression, and depicted as fold of induction to the NT condition. Data in graphs depicts three independent experiments. **(C)** FL-DCs generated from ERAI mice were left untreated (NT) or stimulated with 25, 50, 100, and 200 μg/ml of MEL for 16 h for the quantification of VenusFP expression. Data in graphs depicts the MFI of cDC1 FL-DC (XCR1^+^) of three independent experiments. **(D)** ERAI FL-DCs were NT or stimulated with 100 μg/ml MEL for 24 h for the quantification of VenusFP expression. Data in graphs depicts the MFI of cDC1 FL-DC (XCR1^+^), cDC2 FL-DC (SIPRα^+^), and pDC FL-DC (B220^+^). **(E)** ERAI FL-DCs were NT or stimulated with 100 ug/ml MEL, 100 ug/ml human leukocyte cell lysate or 100 ug/ml B16F10 murine melanoma cell lysates (B16 lysate) for 24 h to evaluate VenusFP expression. Data in graphs depicts the MFI of cDC1 FL-DC (XCR1^+^) of three independent experiments. **(F)** FL-DCs were left untreated (NT) or stimulated with 100 μg/ml B16 lysate or 1 μg/ml TM for 8 h and expression of XBP-1s was measured by qPCR. Data in graphs depicts three independent experiments. **(G)** GMCSF BMDCs were left untreated (NT) or stimulated with 100 μg/ml B16 lysate or 1 μg/ml TM for 8 h and expression of *XBP-1s, BiP*, and *CHOP* mRNA was measured by qPCR relative to *L27* expression, and depicted as fold of induction to the NT condition. Data in graphs show two independent experiments. For **(C**–**E)**, each symbol in the graph represents data derived from one independent experiment. For all error bars represent mean ± SEM. *p < 0.05, **p < 0.01, ***p < 0.001, ****p < 0.0001 (paired Student's *t*-test).

Previous reports have demonstrated that IRE1α activation is a key regulator of cDC1 function and survival in steady state (21, 22). In inflammation, it has been shown that myeloid cells activate the IRE1α/XBP1s axis in response to microbial ligands of Toll-Like Receptors (TLR), RIG-I-like receptors but also with molecules expressed by tumors (25–29, 32, 33). In this context, we sought to investigate if DCs differentially activate the IRE1α/XBP1s axis during recognition of innate stimuli of diverse origin. For this purpose, we generated *in vitro* cultures of bone marrow (BM) cells cultured in presence of the cytokine FLT3L, which is a culture that generates an heterogeneous mix of cell equivalents of cDC1, cDC2, and pDCs (referred to as “FL-DCs”) (Supplemental Figure 1B) (10). We included lipopolysaccharide (LPS) as a microbial stimulus, house dust mite extract (HDM) as a model allergen, and a cell lysate generated from human melanoma cell lines (referred to as “MEL”), as a tumor-related stimulus. MEL has proven to be a clinically effective stimulus in DC vaccines in patients with advanced melanoma, and it is generated by cycles of freeze-thaw of three established human melanoma cell lines (34, 35). We investigated whether LPS, HDM, or MEL lysates induced the splicing of *Xbp1* mRNA (*Xbp1s*) by FL-DCs (Figure 1A). As a positive control we included the pharmacologic ER-stress inducer tunicamycin (TM). Data in Figure 1A shows that MEL lysate preferentially induced *Xbp1s* mRNA in FL-DCs compared to LPS and HDM, a feature that was also observed in qPCR analysis (Figure 1B). The cancer cell lysate also induced expression of additional targets of the UPR in FL-DCs such as the ER chaperone *BiP* (Figure 1B) and showed a trend in the induction of *CHOP*, a transcriptional regulator activated downstream of PERK (Figure 1B). Of note, we confirmed that MEL lysates do not contain viable mRNA that could potentially interfere with these assays (Supplemental Figure 1A). Thus, these data indicate that melanoma cell lysates elicit efficient activation of XBP1s and certain members of the UPR in FL-DCs.

To confirm the activation of the IRE1α arm of the UPR in DC subsets activated with MEL by an independent experimental approach, we generated FL-DCs from the ERAI reporter mice (36). This transgenic mice line reports on IRE1α endonuclease activity by expressing a partial sequence of human XBP1 that includes the IRE1α splicing sites, fused to Venus fluorescent protein (VenusFP) (36). Stimulation of FL-DC cultures from ERAI mice with increasing doses of MEL lysates revealed a dose-dependent effect in the induction of VenusFP in cDC1 equivalents (referred to as “cDC1 FL-DC”) (Figure 1C). However, MEL stimulation also increased VenusFP expression in cDC2 equivalents (referred to as “cDC2 FL-DC”) but not in pDC equivalents (referred to as “pDC FL-DC”) (Figure 1D), demonstrating that only conventional DCs activate IRE1α endonuclease upon MEL recognition.

Next, considering that MEL is a melanoma cell lysate of human origin, we sought to investigate whether the factor driving XBP1s in FL-DCs might also be present in murine melanoma cells. As shown in Figure 1E, stimulation with lysates generated from B16-F10 melanoma cells led to enhanced VenusFP expression in FL-DCs to a similar extent than the human lysates, indicating that the ability to trigger XBP1s is not due to recognition of a xenogeneic factor. Induction of XBP1s by B16 lysates was also confirmed by qPCR analysis (Figure 1F). Furthermore, we also noticed that VenusFP fluorescence in FL-DCs was triggered by melanoma cell lysates but it was not induced by a human-derived blood leukocyte lysate (Figure 1E), suggesting that the factor responsible for XBP1s activation is expressed by cancer cells. Finally, we sought to investigate whether activation of XBP1s triggered by the melanoma lysate was a general feature across DC subtypes. As illustrated in Figure 1G, BMDCs cultured in presence of GMCSF (“GMCSF-BMDCs”), which are an heterogeneous culture of antigen presenting cells phenotypically different to FL-DCs (Supplemental Figure 1B) (37), also induce the expression of *Xbp1s* and *Bip* upon stimulation with B16 lysates, indicating that several DC subtypes can activate the IRE1α/XBP1s axis upon melanoma cell recognition. Altogether, our data indicates that melanoma cell lysates elicit efficient activation of IRE1α endonuclease and *Xbp1s* mRNA in cultures of BMDCs.

### Melanoma Cell Lysates Induce XBP1s, but Not RIDD

**Figure 2:**
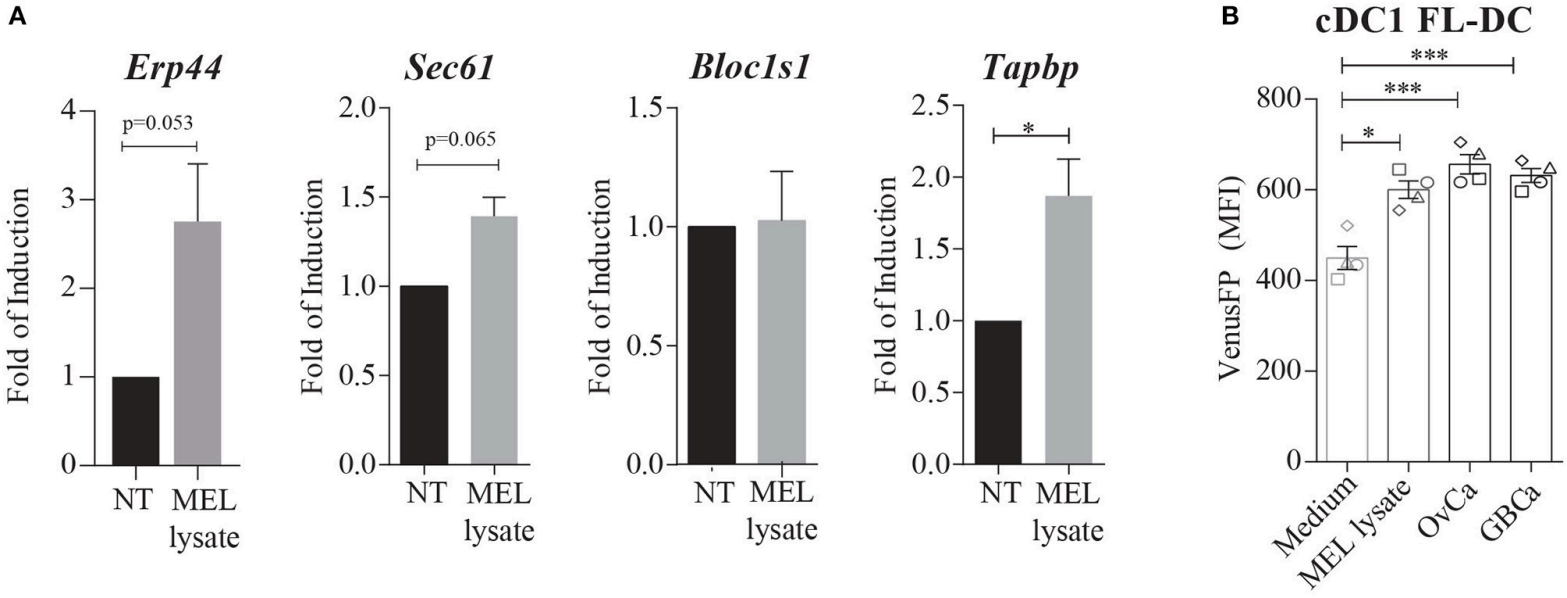
Melanoma cell lysates induce activation of XBP1s and XBP1s-dependent genes, but not RIDD. **(A)** FL-DCs were left untreated (NT) or were stimulated with 100 μg/ml MEL for 8 h. Expression of *Erp44, Sec61a, Bloc1s1*, and *Tapbp* mRNA was measured by qPCR relative to *L27* expression, and depicted as fold of induction to the NT condition. Data in graphs depicts three to five independent experiments. **(B)** Expression of VenusFP in FL-DCs generated from ERAI mice and stimulated with AIM-V medium (control medium) 100 ug/ml MEL, 100 ug/ml human ovarian cancer lysate (OvCa) and 100 ug/ml human gallbladder cancer cells lines (GBCa) for 24 h. Data in graphs depicts the MFI of cDC1 FL-DCs (XCR1^+^) and each symbol in the graph represents data derived from one independent experiment. For all error bars represent mean ± SEM. *p < 0.05, ***p < 0.001 (paired Student's *t*-test).

The ability of melanoma lysates to activate IRE1α and XBP1s prompted us to investigate whether these compounds might also trigger canonical RIDD. Data in Figure 2A illustrates that MEL stimulation in FL-DCs showed a trend in the expression of the XBP1s target gene *Erp44*. The induction of the additional XBP1s target gene *Sec61a* did not reach statistical significance, indicating that MEL lysates do not induce the full XBP1s transcriptional program. Furthermore, MEL-stimulated FL-DCs did not reduce the expression levels of *Bloc1s1*, an archetypical RIDD target or *Tapbp*, a RIDD target in DCs that interferes with the MHC-I antigen presentation pathway (19, 21). These data indicates that RIDD is not induced upon stimulation with melanoma cell lysates. Furthermore, we observed that in addition to MEL, cell lysates generated from ovarian carcinoma cell lines (OvCa) and gallbladder cancer cell lines (GBCa) induced expression of VenusFP in FL-DCs (Figure 2B). Thus, this evidence indicates that lysates derived from various cancer cell types contain factors that induce *Xbp1s* mRNA in DCs.

### Pharmacological Inhibition of IRE1α Endonuclease Decreases the Production of Proinflammatory Cytokines in FL-DCs Stimulated With Tumor Cell Lysates

**Figure 3:**
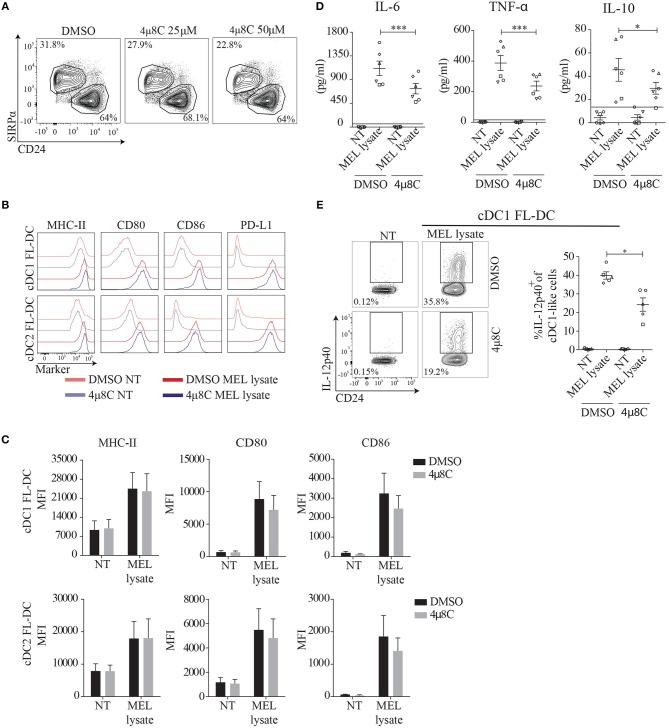
Inhibition of the IRE1α endonuclease domain by the aldehyde 4μ8C does not affect BMDC cellularity or expression of costimulatory molecules, but reduces the production of cytokines upon MEL stimulation. **(A)** FL-DCs were stimulated with increasing doses of 4μ8C or DMSO and composition of DC subtypes was monitored 24 h post treatment. **(B,C)** FL-DCs were pretreated with 20 μM 4μ8C or DMSO for 2 h and stimulated with 100 μg/ml MEL for additional 16 h. Expression of MHC-II, CD80, CD86, and PD-L1 were measured by flow cytometry. Histograms shown in **(B)** are one representative experiment out of five of cDC1 FL-DC (CD24^+^) and cDC2 FL-DC (SIPRα^+^) generated in cultures and graphed in **(C)**. **(D)** FL-DCs were pretreated with 20 μM 4μ8C or DMSO for 5 h and stimulated with 100 μg/ml MEL for additional 16 h. TNF-α, IL-6, and IL-10 were quantified by cytometric bead array. **(E)** FL-DCs were pretreated with 50 μM 4μ8C or DMSO for 5 h and stimulated with 100 μg/ml MEL for additional 16 h. IL-12p40 was analyzed by intracellular staining. Contour plots and graphs are for cDC1 FL-DC (CD24^+^) generated in cultures. For **(D,E)**, each symbol in the graphs represents data derived from one independent experiment. For all error bars represent mean ± SEM. *p < 0.05, ***p < 0.001 (paired Student's *t*-test).

It has been previously reported that IRE1α couples innate recognition with the induction of inflammatory responses (15, 25, 28). To address the contribution of the IRE1α/XBP1s axis in innate recognition of MEL, we used 4μ8C, a selective inhibitor of the IRE1α endonuclease domain (38). Dose titration of 4μ8C in FL-DCs efficiently inhibited XBP1s in response to TM, without affecting survival or overall DC subset composition (Figure 3A and Supplemental Figures 2A,B). To monitor DC maturation, FL-DCs were pre-incubated with 4μ8C or control vehicle and were subsequently stimulated with MEL, and expression of costimulatory molecules was quantified by flow cytometry (Figures 3B,C). Treatment with 4μ8C did not alter surface expression of MHC-II, or the costimulatory molecules CD80, CD86, and PD-L1 in MEL-activated cDC1 and cDC2 FL-DCs (Figures 3B,C). However, we noticed that FL-DCs stimulated with MEL in presence of 4μ8C produced lower levels of the cytokines IL-6, TNF-α, and IL-10 compared to control vehicle (Figure 3D). In addition, the production of IL-12p40, a subunit shared by IL-12 and IL-23, was markedly inhibited by 4μ8C treatment in MEL-stimulated cDC1 FL-DCs (Figure 3E). These data indicates that pharmacological inhibition of IRE1α endonuclease activity in FL-DCs decreases optimal production of IL-6, TNF, IL-10, and IL-12p40 to tumor cell lysates.

### Inhibition of IRE1α Endonuclease Activity Does Not Interfere With Endogenous MHC Class I Presentation and Cross-Presentation in Non-activated FL-DCs

**Figure 4:**
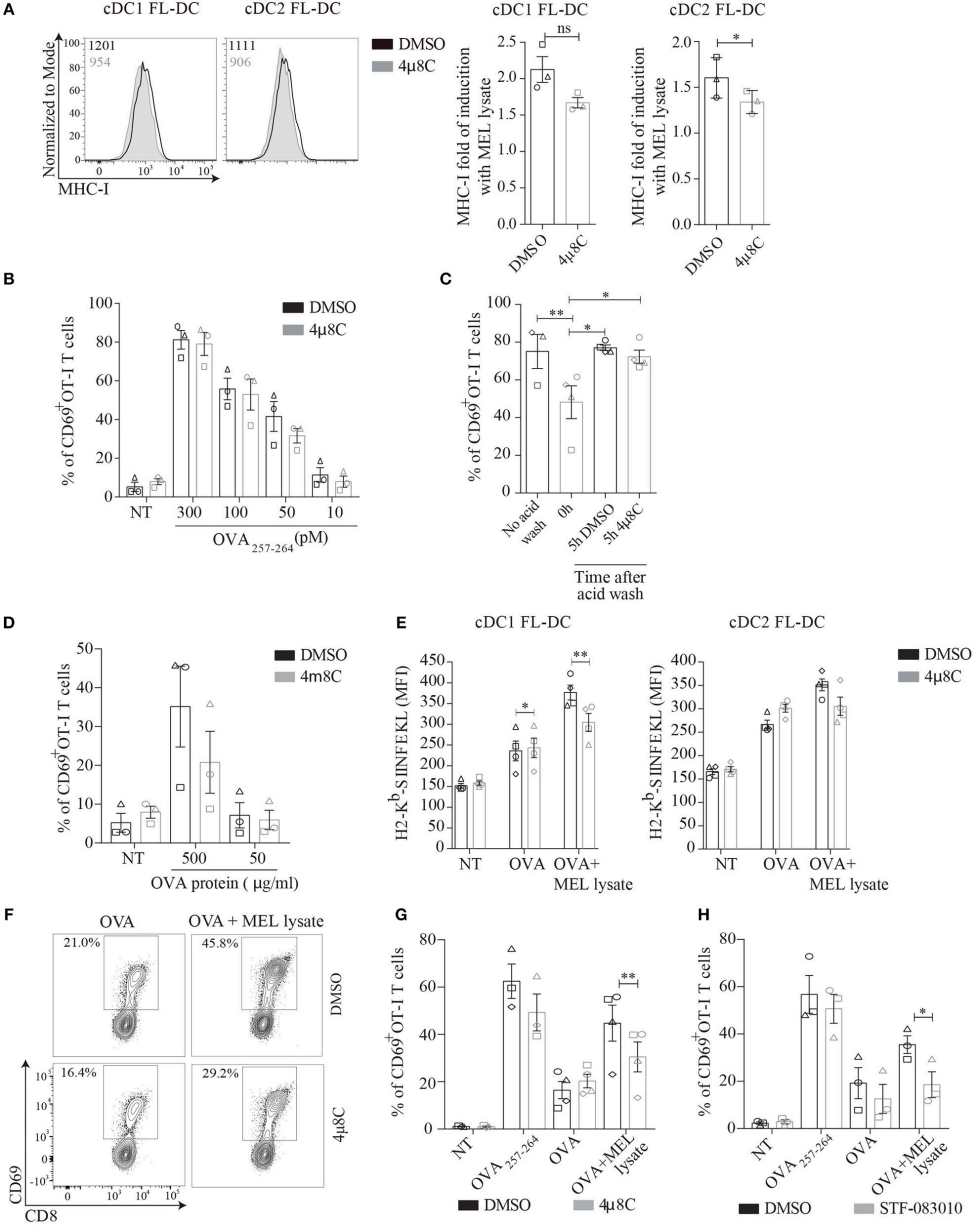
MEL adjuvant function in MHC-I cross-presentation is reduced by inhibition of IRE1α signaling in BMDCs. **(A)** FL-DCs were incubated with 50 μM 4μ8C or DMSO for 6 h and MHC-I expression was measured of cDC1 FL-DC (XCR1^+^) and cDC2 FL-DC (SIPRα^+^) by flow cytometry. Data in graph depicts of three independent experiments. **(B)** FL-DCs were incubated with 50 μM 4μ8C or DMSO for 6 h and then were pulsed with increasing doses of SIINFEKL peptide for the last 20 min of culture. Then cells were counted, fixed and 5 × 10^4^ FL-DCs were cultured with 5 × 10^4^ OT-I T cells. OT-I activation was quantified by expression of CD69. Data in graph shows three independent experiments. **(C)** FL-DCs from DOG mice were incubated with an acid wash solution (see section Materials and Methods) to remove OVA peptides from surface MHC-I molecules. Then cells were incubated in presence of 50 μM 4μ8C or DMSO for 5 h in complete medium and were fixed and cultured with OT-I T cells as in **(B)**. Data is representative of three to four independent experiments. **(D)** FL-DCs were incubated with 50 μM 4μ8C or DMSO for 6 h and then pulsed with increasing concentrations of OVA protein for the last 5 h. Cells were counted, fixed and cultured as in **(B)**. Data in graph shows three independent experiments. **(E)** FL-DCs were incubated with 20 μM 4μ8C or DMSO for 5 h and then stimulated with 250 μg/ml OVA or 250 μg/ml OVA plus 100 μg/ml MEL for 16 h. MHC-I/SIINFEKL complex were measured of cDC1 FL-DC (XCR1^+^) and cDC2 FL-DC (SIPRα^+^) by flow cytometry using 25.D1-16 antibody (H-2K^b^-SIINFEKL). **(F)** FL-DCs were incubated with 50 μM 4μ8C or DMSO for 6 h and then pulsed with 200 μg/ml OVA or 200 μg/ml OVA plus 100 μg/ml MEL for the last 5 h. Alternatively cells were pulsed with 100 pM SIINFEKL peptide for the last 20 min. Cells were counted, fixed and cultured as in **(B)**. **(G)** Data in graph shows three to four independent experiments of **(F)**. **(H)** FL-DCs were incubated with 60 μM STF or DMSO and then treated as in **(B)**. Data in graph shows three independent experiments. Each symbol in the graphs represents data derived from one independent experiment. For all error bars represent mean ± SEM. *p < 0.05, **p < 0.01 (paired Student's *t*-test).

Considering that tumor cells are a relevant source of stimuli for priming cytotoxic T cell responses (39); and that our results indicate that melanoma cell lysates induce the IRE1α/XBP1s axis, we investigated whether this UPR branch could regulate the ability of DCs to activate CD8^+^ T cells upon MEL recognition. To address this issue, we first sought to investigate if acute blockade of IRE1α endonuclease modulated antigen presentation via MHC Class I in resting conditions. This aspect is relevant considering that DCs constitutively activate XBP1s *in vivo* and that genetic ablation of XBP1 in cDC1s leads to the induction of compensatory RIDD in steady state, which prevents the cross-presentation of dead cell-associated antigens (21, 22). Furthermore, it is well-described that genetic ablation of UPR members results in compensatory adaptive mechanisms within the entire UPR pathway (21, 22, 40, 41). We observed that 4μ8C treatment led to a mild reduction in expression of surface levels of MHC Class I, which did not reach significance in cDC1 FL-DCs (Figure 4A). These results prompted to investigate if 4μ8C treatment also resulted in reduced presentation of MHC-I/peptide complexes to CD8^+^ T cells. To this end, FL-DCs were pretreated with 4μ8C- or control vehicle and were subsequently pulsed with various doses of synthetic OVA_257−264_ peptide (which does not require processing by the MHC-I antigen presentation machinery). After the incubation period, cells were fixed and cultured with OT-I T cells (expressing a transgenic, MHC Class I-restricted, TCR specific for OVA_257−264_ derived from ovalbumin, OVA). As shown in Figure 4B, 4μ8C treatment did not affect the ability of FL-DCs to present OVA_257−264_ to OT-I T cells; as measured by expression of the early T cell activation marker CD69. Thus, although 4μ8C treatment resulted in modest reduction of surface MHC-I expression, this effect is not sufficient to inhibit the presentation of specific MHC Class I-peptide complexes leading to T cell activation.

To evaluate if IRE1α via XBP1s modulates the processing route of endogenous antigens in MHC Class I, we generated FL-DCs from CD11c-DOG mice. This is a transgenic mice line that expresses OVA under control of the CD11c promoter, allowing constitutive expression of cytosolic OVA protein in DCs (42). FL-DCs from CD11c DOG mice were treated with acid wash to remove OVA peptides from MHC Class I molecules at the cell surface (43). After treatment with acid wash, cells were allowed to recover for 5 h in presence of 4μ8C or control vehicle and the generation of newly formed MHC Class I/ OVA_257−264_ peptide complexes was quantified upon culture with OT-I T cells (Figure 4C). CD11c DOG FL-DCs that recovered in presence of 4μ8C displayed a similar capacity to activate OT-I cells than cells that recovered in presence of control vehicle. These data indicates that acute blockade of IRE1α endonuclease does not inhibit processing of cytosolic antigens and loading onto MHC Class I molecules (Figure 4C). Finally, to account for cross-presentation in steady state, 4μ8C-treated FL-DCs were pulsed with different doses of soluble OVA protein for 5 h, and cells were fixed and cultured with OT-I cells for quantification of CD69 expression (Figure 4D). No significant differences were observed between 4μ8C treatment and control vehicle in the ability to cross-present soluble OVA protein by resting FL-DCs. Altogether, these data indicates that pharmacological inhibition of IRE1α endonuclease with the aldehyde 4μ8C does not impinge on endogenous MHC Class I presentation and cross-presentation of OVA in absence of innate stimulation.

### Innate Recognition of MEL Lysates Via the IRE1α/XBP1s Axis Favors Cross-Presentation of Antigens to CD8^+^ T Cells

We investigated whether IRE1α activation in response to melanoma cell lysates promoted cross-presentation of OVA. For this purpose, FL-DCs were pre-incubated with 4μ8C or control vehicle and pulsed with OVA or OVA plus MEL and the quantification of MHC-I/OVA OVA_257−264_ peptide complexes was quantified using the antibody 25.D1-16, that recognizes the H-2K^b^-SIINFEKL complex (44) (Figure 4E). No effect of 4μ8C on 25.D1-16 staining was observed in FL-DCs pulsed with OVA alone, in agreement with results shown in Figure 4D. However, in presence of MEL lysates, 4μ8C treatment reduced surface expression of SIINFEKL-loaded MHC-I molecules in FL-DCs, an effect that was particularly noticeable in cDC1 FL-DCs (Figure 4E). These results indicate that pharmacological inhibition of IRE1α endonuclease activity decreases the cross-presentation of MEL-associated antigens. To functionally test for cross-presentation, FL-DCs were incubated with 4μ8C or control vehicle, and pulsed with OVA or OVA plus MEL, and then fixed prior to culture with OT-I T cells (Figures 4F,G). FL-DCs stimulated in presence of MEL-OVA increased the cross-presentation of OVA as indicated by augmented CD69 expression, in comparison with FL-DCs pulsed with OVA in absence of MEL. However, the adjuvant effect of MEL in augmenting OT-I T cell activation was consistently reduced in FL-DCs treated with 4μ8C, suggesting that IRE1α activation upon recognition of MEL lysates favors CD8^+^ T cell activation. Furthermore, to confirm that this effect is specifically attributed to IRE1α activity, we included an additional IRE1α endonuclease inhibitor (STF-083010), which possesses demonstrated *in vivo* activity (45). STF-083010 inhibited XBP1s induced by TM without affecting global viability (Supplemental Figures 2C,D). Similar to the effects noticed with 4μ8C (Figures 4F,G), treatment with STF-083010 also reduced the cross-presentation of OVA by MEL-stimulated FL-DCs (Figure 4H). To sum up, these data indicates that activation of IRE1α endonuclease contributes to decoding the adjuvant effect of MEL lysates for cross-presentation of antigens.

### Inhibition of IRE1α Endonuclease Function Selectively Prevents Cross-Presentation of a Melanoma-Associated Antigen Without Impairing Presentation of Tumor Antigens in MHC Class II

**Figure 5:**
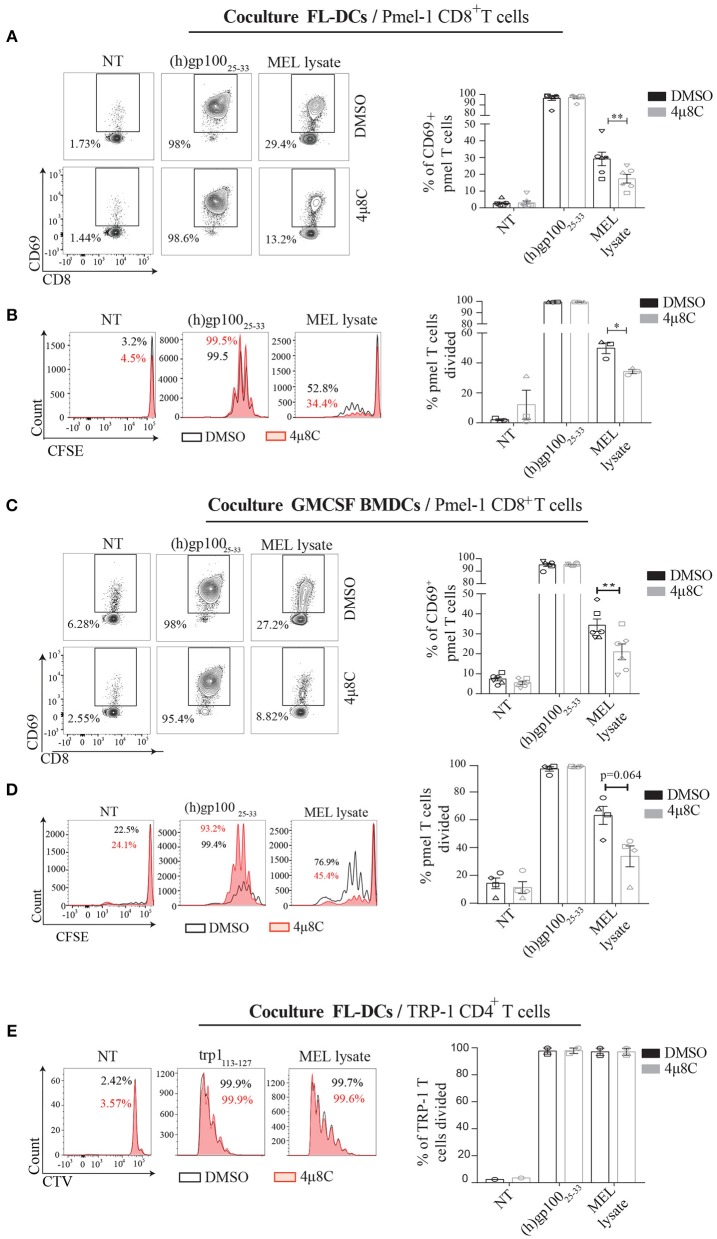
Inhibition of IRE1α endonuclease function reduces the cross-presentation of a melanoma-endogenous antigen *in vitro*. **(A)** FL-DCs were preincubated with 50 μM 4μ8C or DMSO for 6 h and pulsed with 100 μg/ml MEL for the last 5 h of culture. Alternatively, cells were pulsed with 2.5 μM human gp100 peptide for the last 20 min of culture. Cells were counted, fixed and 5 × 10^4^ FL-DCs were cocultured with 5 × 10^4^ pmel-1 CD8^+^ T cells. Pmel-1 CD8^+^ T cell activation was quantified by expression of CD69 on day 1 through flow cytometry. Data in graph shows seven independent experiments. **(B)** FL-DCs were treated as in **(A)** but were not fixed and 2 × 10^4^ FL-DCs were cultured with 5 × 10^4^ CFSE-labeled pmel-1 CD8^+^ T cells. Proliferation was quantified on day 3 by flow cytometry. Data in graph shows three independent experiments. **(C)** GM-CSF BMDCs were treated and cocultured as in **(A)**. Data in graph shows six independent experiments. **(D)** GM-CSF BMDCs were treated and cocultured as in **(B)**. Data in graph shows four independent experiments. **(E)** FL-DCs were treated as in **(B)** but were cultured with 5 × 10^4^ CellTrace Violet-labeled CTV = CD4^+^ T cells isolated from Trp1 mice. Proliferation was measured on day 5 by flow cytometry. Data in graph shows two independent experiments of **(A)**. Each symbol in the graphs represents data derived from one independent experiment. For all error bars represent mean ± SEM. *p < 0.05, **p < 0.01 (paired Student's *t*-test).

To extend our findings to a more physiological setting, we analyzed the cross-presentation of an antigen intrinsic to melanoma cells and investigated the dependence of IRE1α/XBP1s axis in this process. To this end, we isolated CD8^+^ T cells from pmel-1 transgenic mice, which bear a MHC Class I-restricted, transgenic TCR specific for the human and murine melanocyte antigen gp100_25−33_ (46)_._ We verified that MEL lysates contained sufficient amounts of the gp100 antigen, which could only be cross-presented to pmel-1 T cells via a BMDC (Supplemental Figure 3A). Furthermore, we demonstrate that both, MEL lysates and B16 lysates contained antigens for cross-presentation to pmel T cells, showing a higher efficiency for the human lysate over the murine counterpart (Supplemental Figure 3B). These data is consistent with reported work demonstrating that the pmel-1 TCR recognizes the human gp100_25−33_ peptide with greater efficiency than the mouse gp100_25−33_ peptide, due to a more efficient binding of the human sequence to H-2D^b^ (46). These data confirms that MEL lysates are a suitable source of antigen for cross-presentation studies to pmel T cells. We first tested if IRE1α was required for engulfment of MEL lysates, and observed that 4μ8C-treated cells acquire similar amounts of MEL-labeled material over a period of time compared to the control condition (Supplemental Figure 3C), indicating that inhibitor treatment does not affect antigen uptake. Then, we interrogated if MEL-stimulated FL-DCs with an active IRE1α/XBP1s axis were more competent to activate pmel T cells than FL-DCs with the pathway inhibited. Whereas, 4μ8C did not impair MHC Class I presentation of the human gp100_25−33_ peptide, inhibition of IRE1α endonuclease in MEL-stimulated FL-DCs resulted in reduced activation of pmel T cells (Figure 5A). Furthermore, 4μ8C treatment also reduced the ability of MEL-stimulated FL-DCs to trigger pmel T cell proliferation and IFN-γ production (Figure 5B and Supplemental Figure 3D). To extend these findings to additional DC subtypes, we included GMCSF-BMDCs as source of antigen presenting cells and noticed a similar effect than that observed for FL-DCs (Figures 5C,D), indicating that blockade of IRE1α endonuclease activity broadly impacts on the ability of various subtypes of BMDCs to cross-present a melanoma-associated antigen for CD8^+^ T cell activation.

Finally, we investigated if 4μ8C treatment also inhibited the presentation of a melanoma-associated antigen via MHC Class II. To this end, we isolated CD4^+^ T cells from TRP-1 mice, which express a MHC Class II-restricted, transgenic TCR specific for the tyrosinase-related protein 1 antigen present in melanoma (47). In contrast to the observations noticed with pmel CD8^+^ T cells, 4μ8C treatment did not impair the proliferation of TRP-1 CD4^+^ T cells. These data indicates that inhibition of IRE1α endonuclease activity does not influence antigen presentation on MHC Class II (Figure 5E). To sum up, we conclude that activation of the IRE1α/XBP1s axis favors DC activation for CD8^+^ T cell activation to melanoma-associated antigens but it is dispensable for CD4^+^ T cell priming.

### IRE1α Endonuclease Activity Potentiates the Cross-Presentation Abilities of GMCSF-BMDCs *in vivo*

**Figure 6:**
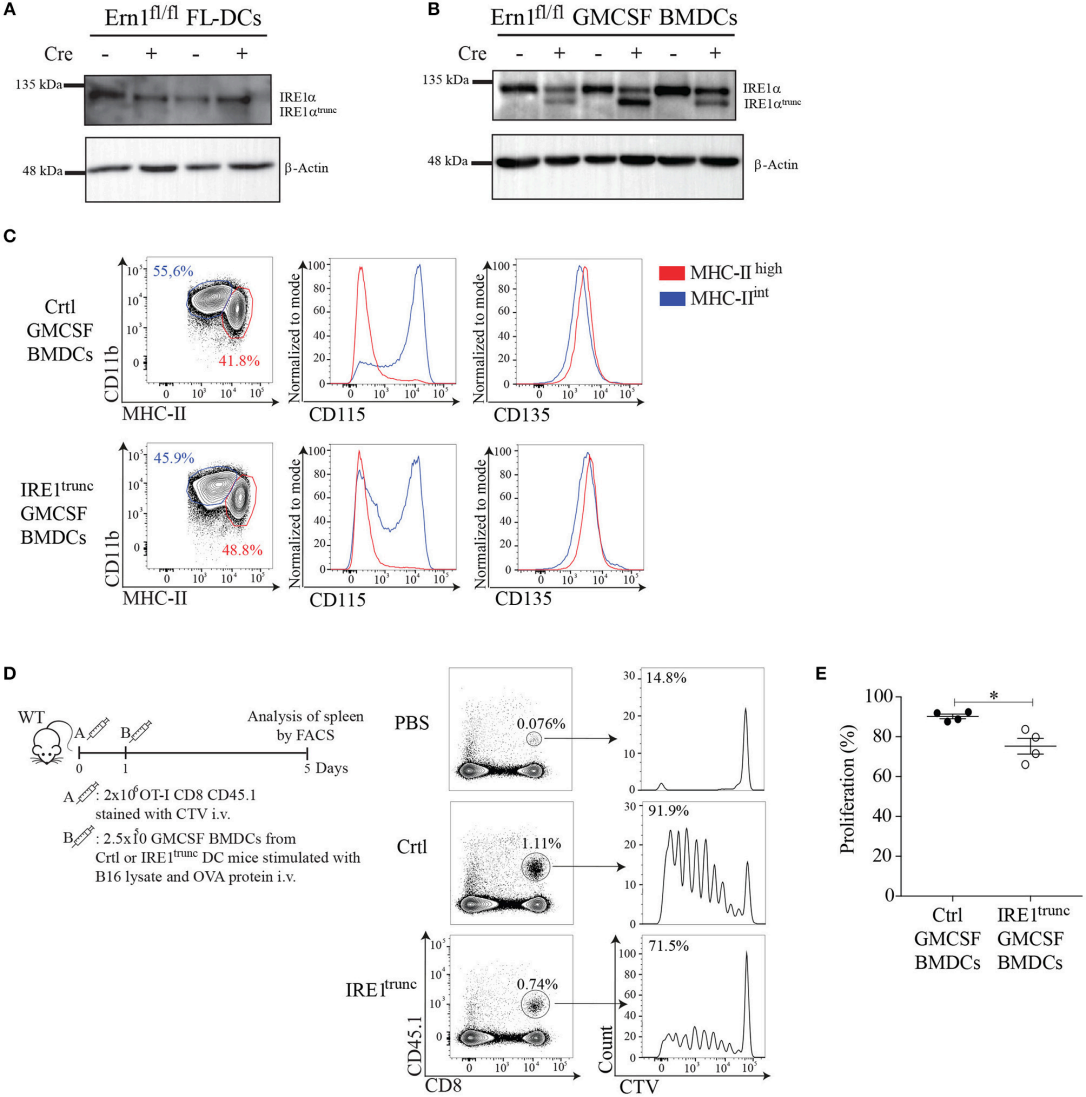
IRE1α endonuclease activity in GMCSF-BMDCs promotes cross-presentation of tumor-associated antigens *in vivo*. **(A,B)** Western Blot analysis of IRE1 levels in FL-DCs and GMCSF BMDCs of IRE1^trunc^ or ctrl DC mice. **(C)** Phenotype of GMCSF BMDCs from IRE1^trunc^ or ctrl DC mice at day 8 of culture (gate on CD11c^+^ cells). **(D)** *In vivo* proliferation of OT-I CD8 T cells (CD45.1^+^). 2 × 10^6^ CD8 T cells stained with CellTrace Violet (CTV) were adoptively transferred into congenic mice. One day later mice were injected i.v. with 2.5 × 10^5^ GMCSF BMDCs, from IRE1^trunc^ or ctrl DC mice pulsed with 100 μg/ml B16 lysate plus 200 μg/ml OVA. Histograms represent the proliferation of transferred cells (CD8^+^ CD45.1^+^) in the spleen. **(E)** The graph represents the percentage of proliferation of CellTrace Violet-labeled cells. Each symbol in the graph represents data from an individual mouse. Error bars represent mean ± S.E.M. *p < 0.05 determined by Mann–Whitney test.

To obtain insights on the function of IRE1α endonuclease activity by an independent approach, we generated DC cultures from BM of IRE1^trunc^ DC mice, which is a crossed mice line between *Itgax*-Cre mice that express Cre recombinase under the promoter of the *Cd11c* gene (48) and *Ern1*^fl/fl^ mice, which have loxP sites flanking exons 20 and 21 of the gene (49). IRE1^trunc^ DC mice harbor a truncated IRE1 isoform that possesses preserved kinase function but impaired endonuclease activity (49). We validated the model by generating FL-DCs and GM-CSF DCs from BM of IRE1^trunc^ DC mice and Ctrl littermates, which correspond to *Ern1*^fl/fl^ mice lacking the Cre recombinase (Figures 6A,B). Remarkably, we observed that FL-DCs from IRE1^trunc^ DC mice do not express the truncated IRE1α isoform and expressed similar amounts of WT IRE1α protein than Ctrl counterparts (Figure 6A). This data indicates that FL-DC cultures do not mediate efficient Cre-dependent excision of the loxP-flanked sites in the *Ern1*^fl/fl^ gene and therefore, are not a suitable model to study loss of IRE1α endonuclease function. However, in cultures of GMCSF-BMDCs from IRE1^trunc^ DC mice, we observed the presence of the truncated IRE1α isoform, although the expression levels of the truncated protein were highly variable among BM cultures derived from independent mice (Figure 6B, line 2,4,6). There was also considerable expression of the WT isoform of IRE1α protein remaining in these cultures, which differs with previous observations with splenic DC counterparts (22). Thus, IRE1^trunc^ GMCSF-BMDCs are a model of DCs expressing a mix of WT and truncated isoforms of IRE1α. We verified that IRE1^trunc^ GMCSF-BMDCs developed normally and that expressed normal levels of CD11c and MHC-II, along with surface markers associated to conventional DCs (CD135, FLT3; receptor for FLT3L) and to monocyte-derived macrophages (CD115), which were previously reported in these cell cultures (37) (Figure 6C).

To test the function of IRE1^trunc^ GMCSF-BMDCs *in vivo*, IRE1^trunc^ or Ctrl cells were stimulated with B16 lysates plus OVA and were then adoptively transferred into B6 mice that receive OT-I T cells labeled with the proliferation dye Cell Trace Violet the day before. OT-I T cell proliferation was monitored on day 5 in spleen (Figure 6D). Adoptive transfer of GMCSF-BMDCs from Ctrl mice elicited a high degree of CD8^+^ T cell activation, as indicated by the proliferation profile of OT-I T cells in spleen. In contrast, adoptive transfer of IRE1^trunc^ GMCSF-BMDCs resulted in a mild but consistent reduction in the frequencies of proliferating OT-I T cells (Figure 6E), which accounted for a 15% reduction in frequencies of proliferating OT-I T cells. These results are consistent with results depicted in Figure 5D and indicate that IRE1α endonuclease function potentiates the cross-presentation of tumor cell associated antigens by *ex-vivo* generated DCs.

## Discussion

The intracellular mechanisms responsible to promote immunogenic DC function in cancer are matter of intense investigation. In this work, we report that recognition of melanoma cell lysates induces efficient activation of the IRE1α/XBP1s axis in BMDCs, which in turn increases cross-presentation of melanoma-associated antigens. Our findings indicate that MEL stimulation induces expression of the canonical UPR member BiP and efficiently triggers XBP1s in absence of RIDD. Further experiments will be necessary to elucidate the nature of the XBP1s-activating factor present in melanoma cell lysates, which is expressed in melanoma cells from human and mice origin, and it is also found in additional cancer cell lines such as ovarian and gallbladder cancer. In this context, it is plausible that activation of the IRE1α/XBP1s axis by MEL occurs downstream of PRR recognition, as it is known that innate immune sensing intersect with the UPR at various points for optimal activation of NF-kB, IRF-3, or JNK (26, 28, 50). On one hand, STING activation couples to the UPR (51) and signaling via TLR2 and TLR4 activate XBP1s via reactive oxygen species (ROS) for exacerbation of cytokine production in macrophages (25). In particular, it has been demonstrated that XBP1s binds to the promoter regions of the *Tnf* and *il-6* genes, providing direct evidence linking the UPR to transcriptional activation of cytokines (25). In fact, most of what is currently known on XBP1s function in the regulation of cytokine production emerges from studies in macrophages (25, 52), and it is not clearly understood if similar mechanisms are applicable to DCs. We observe that pharmacological inhibition of IRE1α endonuclease decreases the production of IL-6, TNF, IL-10, and IL-12p40, by FL-DCs to MEL stimulation, which is reminiscent to data previously reported in XBP1 KO macrophages (25). If TLR-dependent XBP1s activation is a conserved feature across macrophages and DCs, then it would be highly plausible that TLR4 signaling accounted for IRE1α/XBP1s activation in MEL-activated FL-DCs, as it has been previously reported that the melanoma cell lines used in this study express the endogenous TLR4 ligand HMGB1 (34). On a mechanistic basis, it is plausible that XBP1s transcriptionally activate expression of *Tnf* and *Il6* genes, although we do not provide formal evidence of this process in this study. Furthermore, on the basis of the presented experiments, we cannot exclude an XBP1s-independent function of IRE1α endonuclease, as it has been recently reported that the enzyme may degrade certain microRNAs still in absence of canonical RIDD (53). Additional parameters, including upregulation of costimulatory molecules, remained unaffected upon pharmacological blockade of IRE1α endonuclease, indicating that the pathway regulates a particular aspect of the transcriptional program of MEL-activated DCs. Thus, our data shows that the IRE1α/XBP1s axis in BMDCs adjusts the magnitude of cytokine production upon innate recognition of cancer cell lysates.

The endonuclease domain of IRE1α is reported to have dual functions in MHC-I antigen presentation, which may be dependent on the cell lineage, pathological setting or the extent of ER stress that can be tolerated by a particular cell type (41). On one hand, IRE1α via XBP1s has shown to regulate expression of several members of the MHC-I antigen presentation machinery such as calnexin, calreticulin, and Erp57 in HEK 293T^DAX^ cells (54). On the other hand, induction of RIDD in DCs (by means of XBP1 genetic ablation) results in reduced cross-presentation of dead cell-associated antigens *in vivo* (21). Our data shows that acute blockade of IRE1α endonuclease in non-activated FL-DCs does not impair their ability to present cytosolic OVA via MHC-I nor to cross-present OVA protein to OT-I T cells although it modestly reduces surface expression of MHC-I. One possibility accounting for these findings may be that BMDCs express additional regulatory mechanisms to ensure efficient antigen presentation. However, in contexts of DC activation, we demonstrated that XBP1s induction in MEL-stimulated BMDCs promotes their ability to cross-present antigens. Although the magnitude of this response is discrete, it suggests that activation of the IRE1α/XBP1s pathway may be relevant to induce CD8^+^ T cell responses to tumor-derived signals. The intracellular mechanisms by which XBP1s leads to increased cross-presentation of melanoma cell-associated antigens *in vitro* remain to be elucidated, although we show that this effect is independent of antigen uptake and that pharmacological blockade of IRE1α reduces the expression of specific MHC Class I/ peptide complexes at the cell surface.

Importantly, in this work we studied BMDCs from IRE1^trunc^ DC mice. This genetic model of IRE1α endonuclease ablation was proven not to be useful for the study of FL-DCs, which prevented further studies in the cDC1 lineage of DCs. At present it is unclear as to why FL-DCs did not carry out Cre-mediated excision of the *Ern1*-floxed gene but it may be related to the immature stage of FL-DCs found in these cultures (55). Future studies using recently reported protocols for the generation of more authentic cDC1s will be valuable to translate these findings to DC subtypes that may be useful in clinical settings (55). However, in experiments using GMCSF-BMDCs from IRE1^trunc^ mice, which expressed the truncated IRE1α isoform, we noticed that these cells were less competent to induce proliferation of antigen specific CD8^+^ T cells in the spleen. Although this effect was not severe, it is unclear if the presence of a remaining pool of the WT IRE1α isoform noticed in these cultures accounted for the discrete differences. Future studies using additional technologies of genetic editing such as CRISPR-Cas9 could help circumvent this issue and provide a full picture on the role of the pathway in melanoma tumor growth, cytotoxic T cell responses *in vivo* and CD8^+^ T cell memory.

At present, it remains to be further investigated the mechanisms that intersect the IRE1α/XBP1s pathway with the MHC-I antigen presentation and cross-presentation route. In fact, cell biological processes known to enhance the efficiency of cross-presentation such as restraining phagolysosome fusion upon TLR signaling (23) have not been explored as consequence of UPR activation. Future studies will unveil the molecular mechanisms linking the IRE1α arm of the UPR with the MHC-I antigen presentation machinery in contexts of innate recognition.

Finally, an aspect that should not be ignored is that activation of the IRE1α/XBP1s axis in DCs does not predictably lead to enhanced T cell activation. It is reported that XBP1 KO CD11b^+^ DCs infiltrating ovarian cancer tumors are more efficient to activate anti-tumor CD8^+^ and CD4^+^ T cell responses and can control tumor growth (29). Although these and our findings may seem at first glance contradictory, there are aspects to be considered. These include the immunostimulatory or immunosuppressive properties of different cancer cell preparations. This is a highly relevant issue considering that, whereas the conditioned media of ovarian cancer tumors is highly immunosuppressive and curtails T cell proliferation (29), we show that melanoma cell lysates act as adjuvants for cross-presentation. At present, it is not fully understood what dictates the immunogenicity vs. the immunosuppressive properties of preparations from different cancer cell types and in fact, several variables such as the stage of tumor progression, the use of cell lines vs. implanted tumors, the nature of the cancer cell, the amount/type of danger signals expressed by each cancer type could influence this outcome. Furthermore, a possibility is that IRE1α and XBP1s may control different cell biological processes in DCs according to an immunogenic or an immunosuppressive environment. Additional aspects on the role of the IRE1α/XBP1s in promoting tumor cell growth or tumor rejection may also be associated with the extent of ER stress imposed by the tumor microenvironment, which cannot be recapitulated by in *in vitro* approaches. Finally, the functionality of the IRE1α/XBP1s axis in different DC lineages may also play a role, considering that not all tumors are able to recruit the DC subtypes responsible to mediate cytotoxic responses *in vivo*. This is relevant considering that cDC2s, in contrast to cDC1s, are not sensitive to XBP1 loss in resting conditions (21). In the present study, we present evidence that are consistent with previous data showing that enforced XBP1s expression potentiates antitumor T cell immunity of DC vaccines generated *ex vivo* (30, 31). Altogether, our findings support the notion that activation of the IRE1α/XBP1s pathway may be relevant for improving the immunogenic efficacy of DC-based vaccines in melanoma.

## Materials and Methods

### Mice

Wild-type C57BL/6, *Itgax*-Cre mice (48), *Ern1*^fl/fl^ mice (49), *Ern1*^fl/fl^ x *Itgax*-Cre mice (IRE1^trunc^ DC mice), Pmel-1 (46), Trp-1 mice (47), and ERAI mice (36) were bred at Universidad de Chile. OT-I mice (56) CD11c.DOG mice (42) were bred at Fundación Ciencia & Vida. All mice were on a C57BL/6 background and Trp-1 mice were on a RAG^−/−^ background. For all experiments, mice between 5 and 20 weeks of age were bred in specific pathogen-free conditions. All animal experiments were performed in accordance with institutional guidelines for animal care and were approved by the Ethical Review Committees at University of Chile and Fundación Ciencia & Vida.

### Medium and Reagents

Culture medium was RPMI 1640 GlutaMAX™ (Gibco) supplemented with penicillin, streptomycin (Hyclone), 2-mercaptoethanol (Gibco) and 10% heat-inactivated fetal bovine serum (FBS) (Corning). FACS Buffer was PBS 1X (Gibco), supplemented with 1% FBS and 2 mM EDTA (Ambion). Cytometric bead array (CBA) Mouse Inflammation Kit was purchased from BD Biosciences. IRE1 Inhibitor III, 4μ8C (38) was from EMD Millipore. STF-083010 (45), Tunicamycin (TM), lipopolysaccharide (LPS), PMA and Ionomycin were from Sigma-Aldrich. House Dust Mite (*D. pteronyssinus*) was purchased from GreerLabs. OVA_257−264_ peptide (SIINFEKL) was purchased from Invivogen. Soluble Low Endo Ovalbumin was purchased from Worthington Biochemical. Human gp100 peptide (hgp100_25−33_, KVPRNQDWL) and Mouse TRP-1 peptide (TRP-1_106−130_, SGHNCGTCRPGWRGAACNQKILTVR) were purchased from Genetel Laboratories LLC. Brefeldin A was from eBiosciences.

### Cell Lines, Melanoma Lysates, and Supernatants

The human melanoma lysates (MEL) was derived from 3 allogeneic melanoma cell lines (Mel1, Mel2, and Mel3), which were isolated and purified from metastasic lymph nodes (35). Identity of cell lines was confirmed by Short Tandem Repeat (STR) DNA profiling analysis (not shown). Briefly, the lysates were made from a mix of equal amounts of cell lines, taken to a final concentration of 4 × 10^6^ cells/ml, in eppendorf tubes. Cells were lysed through 3 cycles of freeze–thaw in liquid nitrogen. The protein concentration was estimated by Bradford's method using a biophotometer (Eppendorf). The human gallbladder cancer lysates (GBCa) (57), human ovarian cancer cell lysates from SKOV3 cell lines (ATCC) (OvCa), leukocyte lysed from PBMC and B16.F10 cell line lysate (B16 lysate) were lysed using the same method.

### Flow Cytometry and Cell Sorting

Antibodies for flow cytometry were purchased from BD Pharmigen, BD Horizon™, eBioscience, Biolegend or Miltenyi Biotec and the viability dye LIVE/DEAD® Fixable Aqua (Thermofisher Scientific) was used for discriminating dead cells from analysis. Depending on the experiment, cells were stained with the following antibodies in presence of CD16/31 (Fc Block): CD11b (M170), CD86 (GL-1), I-A/I-E (M5/114.15.2), XCR1 (ZET), CD80 (16-10A1), PD-L1 (MIH5), CD8α (53.6.7), CD172α (P84), CD3ε (145-2C11), B220 (RA3-6B2), CD103 (2E7), CD11c (N418), CD69 (IM7), H-2K^b^ (AF6-88.5), CD115 (AFS98), CD24 (M1/49), CD45.1 (A20), CD135 (A2F10), and Streptavidin. Acquisition and analysis of labeled cell suspensions was performed on FACSVerse and LSR Fortessa (BD Biosciences) and subsequent analysis of data was made with FlowJo10 software (FlowJo, LLC). Cell sorting was performed on FACS Aria III (BD Biosciences).

### Generation of Mouse Flt3L and GM-CSF BMDCs

BMDCs were differentiated from femurs and tibias of C57BL/6 mice. FL-DCs (10) were generated by culturing BM cells in culture media in the presence of 150 ng/ml of human recombinant Flt3L (Peprotech) for 7–8 days. GM-CSF DCs (58) were generated by culturing BM cells in the presence of 20 ng/ml mouse recombinant GM-CSF (Biolegend) for 8 days. Fresh culture medium with cytokine was added on day 3, and on day 6 the medium was refreshed.

### BMDCs Activation

2 × 10^5^ FL-DCs were pretreated with 20 μM 4μ8C or DMSO for 2 h and stimulated with 100 μg/ml MEL for 16 h. Expression of MHC-II, CD80, CD86, and PD-L1 was measured by flow cytometry. For CBA, 2 × 10^5^ FLT3-L BMDCs were incubated for 6 h with DMSO or 4μ8C 20 μM, and then stimulated with MEL 100 μg/ml for 16 h. After incubation, cells were centrifuged and supernatant was collected. For activation of ERAI FL-DCs, 2 × 10^5^ cells were stimulated with 100 μg/ml of the following lysate preparations: MEL, B16 lysate, Leukocyte lysate, OvCa, and GBCa for 24 h. Expression of VenusFP was measured by flow cytometry. For MEL titration, 2 × 10^5^ FL-DCs were not treated or stimulated with increasing amounts (2, 50, 100, and 200 μg/ml) of MEL. Expression of VenusFP was measured after 16 h by flow cytometry. For MHC-I staining, FL-DCs were incubated with 50 μM 4μ8C or DMSO for 6 h and MHC-I expression was measured by flow cytometry.

### Quantification of Cytokine Production

For CBA analysis, 2 × 10^5^ Flt3L BMDCs were incubated for 22 h with 20 μM 4μ8C or DMSO, and stimulated with MEL 100 μg/ml for the last 16 h of culture. After incubation, supernatant was collected for cytokine analysis. For intracellular staining of the IL-12p40 subunit, 2 × 10^5^ FL-DCs were stimulated with 50 μM 4μ8C or DMSO at 37°C for 22 h, followed by stimulation with 100 μg/ml of MEL lysates for the last 16 h of culture. During the last 4 h of stimulation, Golgi Plug 1X (BD Biosciences) was added to the wells. After extracellular staining, BMDCs were fixed and permeabilized using the Cytofix/Cytoperm™ fixation/permeabilization kit (BD Biosciences). For IL-12p40 staining, cells were labeled with the IL-12/IL-23 p40 eFluor® 660 antibody (C17.8; eBioscience). For detection of IFNγ, CD8^+^ T cells were collected on day 3 of coculture and were stimulated with 0.25 μM PMA and BFA 1x for 4 h. After extracellular staining, T cells were fixed and permeabilized with the Foxp3/Transcription Factor Fixation/Permeabilization Kit (eBioscience), and cells were labeled with IFNγ PE (XMG1.2, eBioscience).

### PCR, qPCR, and Primers

RNA was obtained from Flt3L BMDCs using the TriPure isolation reagent (Roche, Sigma Aldrich) following the manufacturer's instructions. Complementary DNA (cDNA) was made using the M-MLV Reverse Transcriptase kit (Invitrogen, Thermo Fischer Scientific) and SYBR green-based qPCR was performed using MX3005P (Stratagene, Agilent Techonologies). XBP-1 splicing analysis by conventional PCR as described previously (59). Briefly, cDNA was amplificated and PCR products were digested with the restriction enzyme PstI (Promega) for 2 h and then analyzed in a 1% agarose gel.

For qPCR analysis, BMDCs were treated with medium or stimulated with 100 μg/ml MEL, 100 μg/ml B16 lysate, 100 ng/ml LPS, 50 mg/ml HDM, or 1 μg/ml TM or DMSO ctrl for 8 h. Primers for Sec61 and XBP-1 were from Lee et al. (40), primers for Erp44, Bloc1s1, and Tapbp were from Osorio et al. (21). Other qPCR primers used in this study were from Roche Universal Probe Library: Bip forward (5′-ATGAGGCTGTAGCCTATGGTG-3′); Bip reverse (5′-GGGGACAAACATCAAGCAG-3); CHOP forward (5'-CCACCACACCTGAAAGCAG′-3′); CHOP reverse (5′-TCCTGCAGATCCTCATACCAG-3′); L27 forward (5′-GCCAAGCGATCCAAGATCAA-3′); L27 reverse (5′-GCTGGGTCCCTGAACACATC-3′).

### Antigen Presentation Assays

CD8^+^T cells were isolated from spleen of OT-I or Pmel-1 mice, while CD4^+^ T cells were isolated from spleen and lymph nodes of Trp-1 mice. CD8^+^ T Cells were isolated by negative selection using a lineage depletion cocktail of biotinylated antibodies and anti-biotin microbeads (Miltenyi Biotec) and labeled with 5 μM CFSE (eBioscience) when described. CD4^+^ T cells were isolated by cell sorting gating on FSC/SSC/singlets/CD3^+^/CD4^+^ and labeled with 5 μM CellTrace™ Violet (CTV) (Thermofisher). BMDCs were treated with 50 μM 4μ8C or 60 μM STF-083010 or DMSO as vehicle control. One hour later, OVA (200 μg/ml) and/or MEL lysates (100 μg/ml) were added to the wells containing the inhibitors and cells were incubated for 5 additional hours. For MHC-I presentation of peptides, BMDCs were pulsed for the last 20 min of culture with the following peptides OVA_257−264_ (300, 100, 50, or 10 pM); hgp100_25−33_ (2.5 μM), TRP-1_106−130_ (2.5 μM). For assays measuring early T cell activation, DCs were collected, washed with FACS buffer and fixed with PFA 1% for 10 min. Then cells were washed with 0.2 M glycine and were washed with media prior to coculture. 5 × 10^4^ fixed DCs were cultured with 5 × 10^4^ T cells (1:1 ratio) at 37°C for 16 h to analyse T cell activation by flow cytometry by means of CD69 expression. For proliferation assays, DCs were pulsed with inhibitors and antigens as described above with the exception that cells were not fixed at the end of the culture. 2 × 10^4^ DCs were cultured with 5 × 10^4^ CFSE o CTV labeled T cells for 3 days and proliferation was measured by flow cytometry.

### Endogenous MHC-I Presentation Assay

BM from CD11c.DOG mice was used to generate FL-DCs as described. On day 8, DCs were centrifuged and incubated for at 4°C for 2 min with citric acid (Acid wash solution, pH = 3.94), 1% BSA to remove constitutive OVA peptides from surface MHC class I molecules (60). After the incubation, cells were washed 3 times with complete culture media. Then, DCs were allowed to recover in presence of 50 μM 4μ8C or DMSO control for 5 h in complete media at 37°C. Cells were then fixed in PFA and were cocultured with purified CD8^+^ OT-I T cells in a 1:1 ratio. CD69 expression on CD8^+^ T cells was measured by flow cytometry after 16 h of culture.

### Phagocytic Uptake Assay

1 × 10^7^ Mel2 cells were washed with un-supplemented RPMI (Corning) and stained with 2 μM PKH26 membrane linker solution (Sigma-Aldrich) following manufacturer's instructions. Cells lysates of PKH26 labeled cells were generated as previously described. For phagocytic uptake, FL-DCs were incubated with 50 μM 4μ8C or DMSO, in presence of PKH26 labeled MEL in a 2:1 Tumor cell: DC ratio for 0, 60, and 120 min at 37 or 4°C as control of phagocytosis. Internalization of PKH26 labeled material by DCs was assessed by flow cytometry, gating on the DC population.

### H-2K^b^-SIINFEKL Staining

2 × 10^5^ FL-DCs per condition were incubated with 20 μM 4μ8C or DMSO for 5 h and then not treated or stimulated with 250 μg/ml OVA or 250 μg/ml OVA plus 100 μg/ml MEL for 16 h. Cells were collected and incubated at 4°C for 1 h with H-2K^b^-SIINFEKL PE-Cy7 antibody (25.D1-16; Biolegend) in FACS Buffer. Then antibody cocktail plus Fc Block 2X was added and incubated at 4°C for 20 min in FACS Buffer. MHC-I/SIINFEKL complex were measured by flow cytometry.

### Western Blot

BMDCs were spun at 400 g for 7 min, the supernatant was removed and the pellet resuspended in ice-cold PBS. After a next round of centrifugation (400 g, 7 min), the pellet was pipetted dry and resuspended in 30 or 50 μl of E1A buffer (1% NP40, 20 mM HEPES, pH 7.9, 250 mM NaCl, 1 mM EDTA) complemented with Complete-ULTRA (Roche) and PhosSTOP (Roche). Samples were incubated in buffer at 4°C for 15 min, vortexing every 5 min, then spun at 12,000 g to remove insoluble material and stored at −80°C until further use. Prior to SDS–PAGE, samples were resuspended in loading dye and heated at 95°C for 10 min. After wet transfer to polyvinyldifluoride membrane (Immobilon; Millipore), proteins were analyzed by immunoblotting and visualized by chemiluminescence (Luminata Forte Western HRP substrate; Millipore). Antibodies used recognize IRE1α (Rabbit 14C10; Cell Signaling; used 1/1,000), β-Actin (Mouse ab6276; Abcam; used 1/5,000); Secondary antibodies Anti-Rabbit (Cell signaling; used 1/4,000), Anti-Mouse (Cell signaling; used 1/4,000).

### *In vivo* Proliferation Assay

For *in vivo* proliferation assay, 2 × 10^6^ OT-I CD8 T cells (CD45.1^+^) stained with CTV were intravenously transferred (i.v.) into CD45.2 congenic mice. Next day, mice were injected i.v. with 2.5 × 10^5^ GMCSF BMDCs from IRE1^trunc^ or control DC mice stimulated for 16 h with 100 μg/ml B16- F10 lysates plus 200 μg/ml OVA. Four days later, the proliferation of transferred cells was measured in the spleen by flow cytometry.

### Statistical Analysis

Differences between groups were analyzed by paired, two-tailed Student's *t*-tests or Mann–Whitney test. Results with a *P*-value of 0.05 or less were considered significant. Mean values, SEM and statistics were calculated using Graphpad Prism Software. ^*^p < 0.05, ^**^p < 0.01, ^***^p < 0.001. No criteria of inclusion/exclusion of data were used in this study.

## Author Contributions

CC, BM, and DF conducted experiments. CP managed cell lines and generated cell lysates. AL, DS, and RP provided critical reagents. FS-O contributed with critical discussions and key reagents. TI provided ERAI reporter mice and *Ern1*^fl/fl^ mice. CC, BM, DF, and FO designed the study, analyzed the data, and wrote the manuscript.

### Conflict of Interest Statement

The authors declare that the research was conducted in the absence of any commercial or financial relationships that could be construed as a potential conflict of interest.

## Abbreviations

ATF6: activating transcription factor 6
BM: bone marrow
DC: dendritic cell
cDC: conventional DC
cDC1: conventional DC type 1 (XCR1^+^ or CD24^+^ DC)
cDC2: conventional DC type 2 (SIRPα^+^ DC)
ER: endoplasmic reticulum
ERAI: ER stress-activated indicator
Flt3L: FMS-related tyrosine kinase 3 ligand
FP: fluorescent protein
GM-CSF: granulocyte macrophage colony-stimulating factor
IRE1: inositol-requiring enzyme 1
KO: Knock-out
MEL: Human melanoma cell line lysates
MHC class I: major histocompatibility class I
pDC: plasmacytoid DC
PERK: protein kinase R-like ER kinase
RIDD: regulated IRE1-dependent decay
TRP-1: Tyrosinase-Related Protein 1
UPR: unfolded protein response
XBP1s: spliced XBP1
XBP1u: unspliced XBP1.

## References

[1] Steinman RM. Dendritic cells: versatile controllers of the immune system. Nat Med. (2007) 13:1155–9. 10.1038/nm1643 17917664

[2] Guilliams M Ginhoux F Jakubzick C Naik SH Onai N Schraml BU. Dendritic cells, monocytes and macrophages: a unified nomenclature based on ontogeny. Nat Rev Immunol. (2014) 14:571–8. 10.1038/nri3712 25033907 PMC4638219

[3] Crozat K Guiton R Contreras V Feuillet V Dutertre C-A Ventre E. The XC chemokine receptor 1 is a conserved selective marker of mammalian cells homologous to mouse CD8alpha^+^ dendritic cells. J Exp Med. (2010) 207:1283–92. 10.1084/jem.20100223 20479118 PMC2882835

[4] Durai V Murphy KM. Functions of murine dendritic cells. Immunity (2016) 45:719–36. 10.1016/j.immuni.2016.10.010 27760337 PMC5145312

[5] Merad M Sathe P Helft J Miller J Mortha A. The dendritic cell lineage: ontogeny and function of dendritic cells and their subsets in the steady state and the inflamed setting. Annu Rev Immunol. (2013) 31:563–604. 10.1146/annurev-immunol-020711-074950 23516985 PMC3853342

[6] Poulin LF Reyal Y Uronen-Hansson H Schraml BU Sancho D Murphy KM. DNGR-1 is a specific and universal marker of mouse and human Batf3-dependent dendritic cells in lymphoid and nonlymphoid tissues. Blood (2012) 119:6052–62. 10.1182/blood-2012-01-406967 22442345

[7] Theisen D Murphy K. The role of cDC1s *in vivo*: CD8 T cell priming through cross-presentation. F1000 Res. (2017) 6:98. 10.12688/f1000research.9997.1 28184299 PMC5288679

[8] Nierkens S Tel J Janssen E Adema GJ Antigen cross-presentation by dendritic cell subsets: one general or all sergeants? Trends Immunol. (2013) 34:361–70. 10.1016/j.it.2013.02.007 23540650 PMC4351710

[9] Inaba K Inaba M Romani N Aya H Deguchi M Ikehara S. Generation of large numbers of dendritic cells from mouse bone marrow cultures supplemented with granulocyte/macrophage colony-stimulating factor. J Exp Med. (1992) 176:1693–702. 10.1084/jem.176.6.1693 1460426 PMC2119469

[10] Naik SH Proietto AI Wilson NS Dakic A Schnorrer P Fuchsberger M. Cutting edge: generation of splenic CD8^+^ and CD8^−^ dendritic cell equivalents in Fms-like tyrosine kinase 3 ligand bone marrow cultures. J Immunol. (2005) 174:6592–7. 10.4049/jimmunol.174.11.6592 15905497

[11] Briseño CG Haldar M Kretzer NM Wu X Theisen DJ Kc W. Distinct transcriptional programs control cross-priming in classical and monocyte-derived dendritic cells. Cell Rep. (2016) 15:2462–74. 10.1016/j.celrep.2016.05.025 27264183 PMC4941620

[12] Palucka K Banchereau J. Dendritic-cell-based therapeutic cancer vaccines. Immunity (2013) 39:38–48. 10.1016/j.immuni.2013.07.004 23890062 PMC3788678

[13] Pulendran B. The varieties of immunological experience: of pathogens, stress, and dendritic cells. Annu Rev Immunol. (2015) 33:563–606. 10.1146/annurev-immunol-020711-075049 25665078

[14] Janssens S Pulendran B Lambrecht BN. Emerging functions of the unfolded protein response in immunity. Nat Immunol. (2014) 15:910–9. 10.1038/ni.2991 25232821 PMC4388558

[15] Grootjans J Kaser A Kaufman RJ Blumberg RS. The unfolded protein response in immunity and inflammation. Nat Rev Immunol. (2016) 16:469–84. 10.1038/nri.2016.62 27346803 PMC5310224

[16] Hetz C Papa FR. The unfolded protein response and cell fate control. Mol Cell (2018) 69:169–81. 10.1016/j.molcel.2017.06.017 29107536

[17] Tabas I Ron D. Integrating the mechanisms of apoptosis induced by endoplasmic reticulum stress. Nat Cell Biol. (2011) 13:184–90. 10.1038/ncb0311-184 21364565 PMC3107571

[18] Bettigole SE Glimcher LH. Endoplasmic reticulum stress in immunity. Annu Rev Immunol. (2015) 33:107–38. 10.1146/annurev-immunol-032414-112116 25493331

[19] Hollien J Weissman JS. Decay of endoplasmic reticulum-localized mRNAs during the unfolded protein response. Science (2006) 313:104–7. 10.1126/science.1129631 16825573

[20] Iwakoshi NN Pypaert M Glimcher LH. The transcription factor XBP-1 is essential for the development and survival of dendritic cells. J Exp Med. (2007) 204:2267–75. 10.1084/jem.20070525 17875675 PMC2118458

[21] Osorio F Tavernier SJ Hoffmann E Saeys Y Martens L Vetters J. The unfolded-protein-response sensor IRE-1α regulates the function of CD8α^+^ dendritic cells. Nat Immunol. (2014) 15:248–57. 10.1038/ni.2808 24441789

[22] Tavernier SJ Osorio F Vandersarren L Vetters J Vanlangenakker N Van Isterdael G. Regulated IRE1-dependent mRNA decay sets the threshold for dendritic cell survival. Nat Cell Biol. (2017) 19:698–710. 10.1038/ncb3518 28459443 PMC5563826

[23] Alloatti A Kotsias F Pauwels A-M Carpier J-M Jouve M Timmerman E. Toll-like receptor 4 engagement on dendritic cells restrains phago-lysosome fusion and promotes cross-presentation of antigens. Immunity (2015) 43:1087–100. 10.1016/j.immuni.2015.11.006 26682983

[24] Lerner AG Upton J-P Praveen PVK Ghosh R Nakagawa Y Igbaria A. IRE1α induces thioredoxin-interacting protein to activate the NLRP3 inflammasome and promote programmed cell death under irremediable ER stress. Cell Metab. (2012) 16:250–64. 10.1016/j.cmet.2012.07.007 22883233 PMC4014071

[25] Martinon F Chen X Lee A-H Glimcher LH. TLR activation of the transcription factor XBP1 regulates innate immune responses in macrophages. Nat Immunol. (2010) 11:411–8. 10.1038/ni.1857 20351694 PMC3113706

[26] Moretti J Blander JM. Cell-autonomous stress responses in innate immunity. J Leukoc Biol. (2017) 101:77–86. 10.1189/jlb.2MR0416-201R 27733577 PMC6608034

[27] Hu F Yu X Wang H Zuo D Guo C Yi H. ER stress and its regulator X-box-binding protein-1 enhance polyIC-induced innate immune response in dendritic cells. Eur J Immunol. (2011) 41:1086–97. 10.1002/eji.201040831 21400498 PMC3157298

[28] Keestra-Gounder AM Byndloss MX Seyffert N Young BM Chávez-Arroyo A Tsai AY. NOD1 and NOD2 signalling links ER stress with inflammation. Nature (2016) 532:394–7. 10.1038/nature17631 27007849 PMC4869892

[29] Cubillos-Ruiz JR Silberman PC Rutkowski MR Chopra S Perales-Puchalt A Song M. ER stress sensor XBP1 controls anti-tumor immunity by disrupting dendritic cell homeostasis. Cell (2015) 161:1527–38. 10.1016/j.cell.2015.05.025 26073941 PMC4580135

[30] Tian S Liu Z Donahue C Falo LD You Z. Genetic targeting of the active transcription factor XBP1s to dendritic cells potentiates vaccine-induced prophylactic and therapeutic antitumor immunity. Mol Ther J Am Soc Gene Ther. (2012) 20:432–42. 10.1038/mt.2011.183 21934655 PMC3277233

[31] Zhang Y Chen G Liu Z Tian S Zhang J Carey CD. Genetic vaccines to potentiate the effective CD103^+^ dendritic cell-mediated cross-priming of antitumor immunity. J Immunol. (2015) 194:5937–47. 10.4049/jimmunol.1500089 25972487 PMC4458448

[32] Hasnain SZ Lourie R Das I Chen AC-H McGuckin MA. The interplay between endoplasmic reticulum stress and inflammation. Immunol Cell Biol. (2012) 90:260–70. 10.1038/icb.2011.112 22249202 PMC7165805

[33] Martinon F Glimcher LH. Regulation of innate immunity by signaling pathways emerging from the endoplasmic reticulum. Curr Opin Immunol. (2011) 23:35–40. 10.1016/j.coi.2010.10.016 21094031 PMC3042531

[34] Aguilera R Saffie C Tittarelli A González FE Ramírez M Reyes D. Heat-shock induction of tumor-derived danger signals mediates rapid monocyte differentiation into clinically effective dendritic cells. Clin Cancer Res. (2011) 17:2474–83. 10.1158/1078-0432.CCR-10-2384 21292818

[35] López MN Pereda C Segal G Muñoz L Aguilera R González FE. Prolonged survival of dendritic cell-vaccinated melanoma patients correlates with tumor-specific delayed type IV hypersensitivity response and reduction of tumor growth factor β-expressing T cells. J Clin Oncol. (2009) 27:945–52. 10.1200/JCO.2008.18.0794 19139436

[36] Iwawaki T Akai R Kohno K Miura M. A transgenic mouse model for monitoring endoplasmic reticulum stress. Nat Med. (2004) 10:98–102. 10.1038/nm970 14702639

[37] Helft J Böttcher J Chakravarty P Zelenay S Huotari J Schraml BU. GM-CSF mouse bone marrow cultures comprise a heterogeneous population of CD11c^+^MHCII^+^ macrophages and dendritic cells. Immunity (2015) 42:1197–211. 10.1016/j.immuni.2015.05.018 26084029

[38] Cross BCS Bond PJ Sadowski PG Jha BK Zak J Goodman JM. The molecular basis for selective inhibition of unconventional mRNA splicing by an IRE1-binding small molecule. Proc Natl Acad Sci USA. (2012) 109:E869–78. 10.1073/pnas.1115623109 22315414 PMC3326519

[39] Kroemer G Galluzzi L Kepp O Zitvogel L Immunogenic cell death in cancer therapy. Annu Rev Immunol. (2013) 31:51–72. 10.1146/annurev-immunol-032712-100008 23157435

[40] Lee A-H Scapa EF Cohen DE Glimcher LH. Regulation of hepatic lipogenesis by the transcription factor XBP1. Science (2008) 320:1492–6. 10.1126/science.1158042 18556558 PMC3620093

[41] Osorio F Lambrecht BN Janssens S. Antigen presentation unfolded: identifying convergence points between the UPR and antigen presentation pathways. Curr Opin Immunol. (2018) 52:100–7. 10.1016/j.coi.2018.04.020 29754111

[42] Hochweller K Striegler J Hämmerling GJ Garbi N. A novel CD11c.DTR transgenic mouse for depletion of dendritic cells reveals their requirement for homeostatic proliferation of natural killer cells. Eur J Immunol. (2008) 38:2776–83. 10.1002/eji.200838659 18825750

[43] Cebrian I Visentin G Blanchard N Jouve M Bobard A Moita C. Sec22b regulates phagosomal maturation and antigen crosspresentation by dendritic cells. Cell (2011) 147:1355–68. 10.1016/j.cell.2011.11.021 22153078

[44] Porgador A Yewdell JW Deng Y Bennink JR Germain RN. Localization, quantitation, and *in situ* detection of specific peptide-MHC class I complexes using a monoclonal antibody. Immunity (1997) 6:715–26. 10.1016/s1074-7613(00)80447-1 9208844

[45] Papandreou I Denko NC Olson M Van Melckebeke H Lust S Tam A. Identification of an Ire1alpha endonuclease specific inhibitor with cytotoxic activity against human multiple myeloma. Blood (2011) 117:1311–4. 10.1182/blood-2010-08-303099 21081713 PMC3056474

[46] Overwijk WW Theoret MR Finkelstein SE Surman DR de Jong LA Vyth-Dreese FA. Tumor regression and autoimmunity after reversal of a functionally tolerant state of self-reactive CD8^+^ T cells. J Exp Med. (2003) 198:569–80. 10.1084/jem.20030590 12925674 PMC2194177

[47] Muranski P Boni A Antony PA Cassard L Irvine KR Kaiser A. Tumor-specific Th17-polarized cells eradicate large established melanoma. Blood (2008) 112:362–73. 10.1182/blood-2007-11-120998 18354038 PMC2442746

[48] Caton ML Smith-Raska MR Reizis B. Notch-RBP-J signaling controls the homeostasis of CD8^−^ dendritic cells in the spleen. J Exp Med. (2007) 204:1653–64. 10.1084/jem.20062648 17591855 PMC2118632

[49] Iwawaki T Akai R Yamanaka S Kohno K. Function of IRE1 alpha in the placenta is essential for placental development and embryonic viability. Proc Natl Acad Sci USA. (2009) 106:16657–62. 10.1073/pnas.0903775106 19805353 PMC2757843

[50] Martinon F. Inflammation initiated by stressed organelles. Joint Bone Spine (2017) 85:423–8. 10.1016/j.jbspin.2017.06.005 28705494

[51] Petrasek J Iracheta-Vellve A Csak T Satishchandran A Kodys K Kurt-Jones EA. STING-IRF3 pathway links endoplasmic reticulum stress with hepatocyte apoptosis in early alcoholic liver disease. Proc Natl Acad Sci USA. (2013) 110:16544–9. 10.1073/pnas.1308331110 24052526 PMC3799324

[52] Zeng L Liu Y-P Sha H Chen H Qi L Smith JA. XBP-1 couples endoplasmic reticulum stress to augmented IFN-beta induction via a cis-acting enhancer in macrophages. J Immunol. (2010) 185:2324–30. 10.4049/jimmunol.0903052 20660350 PMC2916979

[53] Fink SL Jayewickreme TR Molony RD Iwawaki T Landis CS Lindenbach BD. IRE1α promotes viral infection by conferring resistance to apoptosis. Sci Signal. (2017) 10:eaai7814. 10.1126/scisignal.aai7814 28588082 PMC5535312

[54] Shoulders MD Ryno LM Genereux JC Moresco JJ Tu PG Wu C. Stress-independent activation of XBP1s and/or ATF6 reveals three functionally diverse ER proteostasis environments. Cell Rep. (2013) 3:1279–92. 10.1016/j.celrep.2013.03.024 23583182 PMC3754422

[55] Kirkling ME Cytlak U Lau CM Lewis KL Resteu A Khodadadi-Jamayran A. Notch signaling facilitates *in vitro* generation of cross-presenting classical dendritic cells. Cell Rep. (2018) 23:3658–72.e6. 10.1016/j.celrep.2018.05.068 29925006 PMC6063084

[56] Clarke SR Barnden M Kurts C Carbone FR Miller JF Heath WR. Characterization of the ovalbumin-specific TCR transgenic line OT-I: MHC elements for positive and negative selection. Immunol Cell Biol. (2000) 78:110–7. 10.1046/j.1440-1711.2000.00889.x 10762410

[57] Rojas-Sepúlveda D Tittarelli A Gleisner MA Ávalos I Pereda C Gallegos I. Tumor lysate-based vaccines: on the road to immunotherapy for gallbladder cancer. Cancer Immunol Immunother. (2018) 67:1897–910. 10.1007/s00262-018-2157-5 29600445 PMC6244977

[58] Lutz MB Kukutsch N Ogilvie ALJ Rößner S Koch F Romani N. An advanced culture method for generating large quantities of highly pure dendritic cells from mouse bone marrow. J Immunol Methods (1999) 223:77–92. 10.,1016/S0022-1759(98)00204-X 10037236

[59] Calfon M Zeng H Urano F Till JH Hubbard SR Harding HP. IRE1 couples endoplasmic reticulum load to secretory capacity by processing the *XBP-1* mRNA. Nature (2002) 415:92–6. 10.1038/415092a 11780124

[60] Sugawara S Abo T Kumagai K. A simple method to eliminate the antigenicity of surface class I MHC molecules from the membrane of viable cells by acid treatment at pH 3. J Immunol Methods (1987) 100:83–90. 10.1016/0022-1759(87)90175-x 3298442

