# Supplementary figures and images for: IRE1α Activation in Bone Marrow-Derived Dendritic Cells Modulates Innate Recognition of Melanoma Cells and Favors CD8+ T Cell Priming
Source: Front Immunol. 2019 Jan 4;9:3050. doi: 10.3389/fimmu.2018.03050 (PMC6338037; doi:10.3389/fimmu.2018.03050)

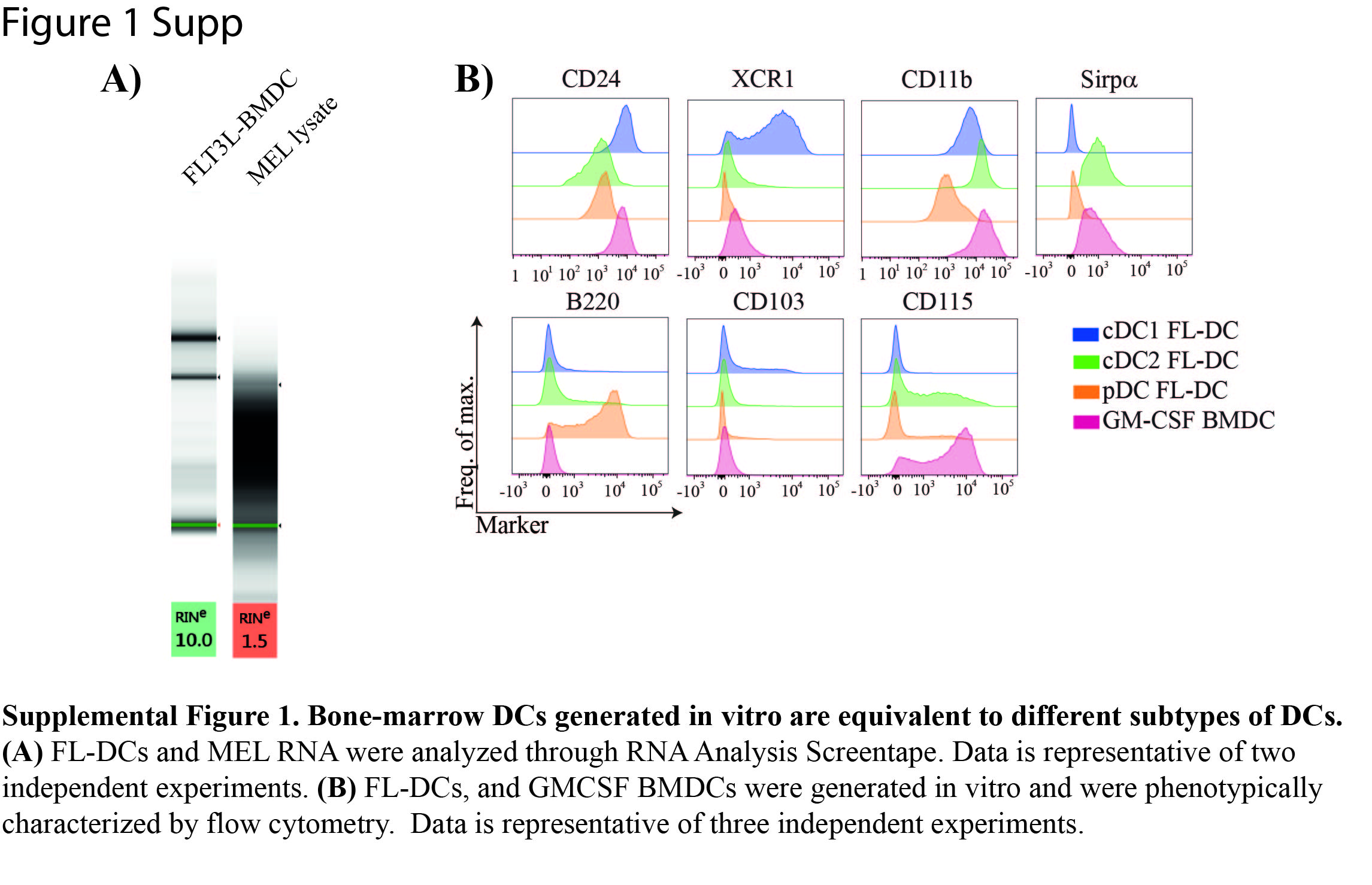

Supplement: Supplementary file 1 [file Image_1.JPEG]

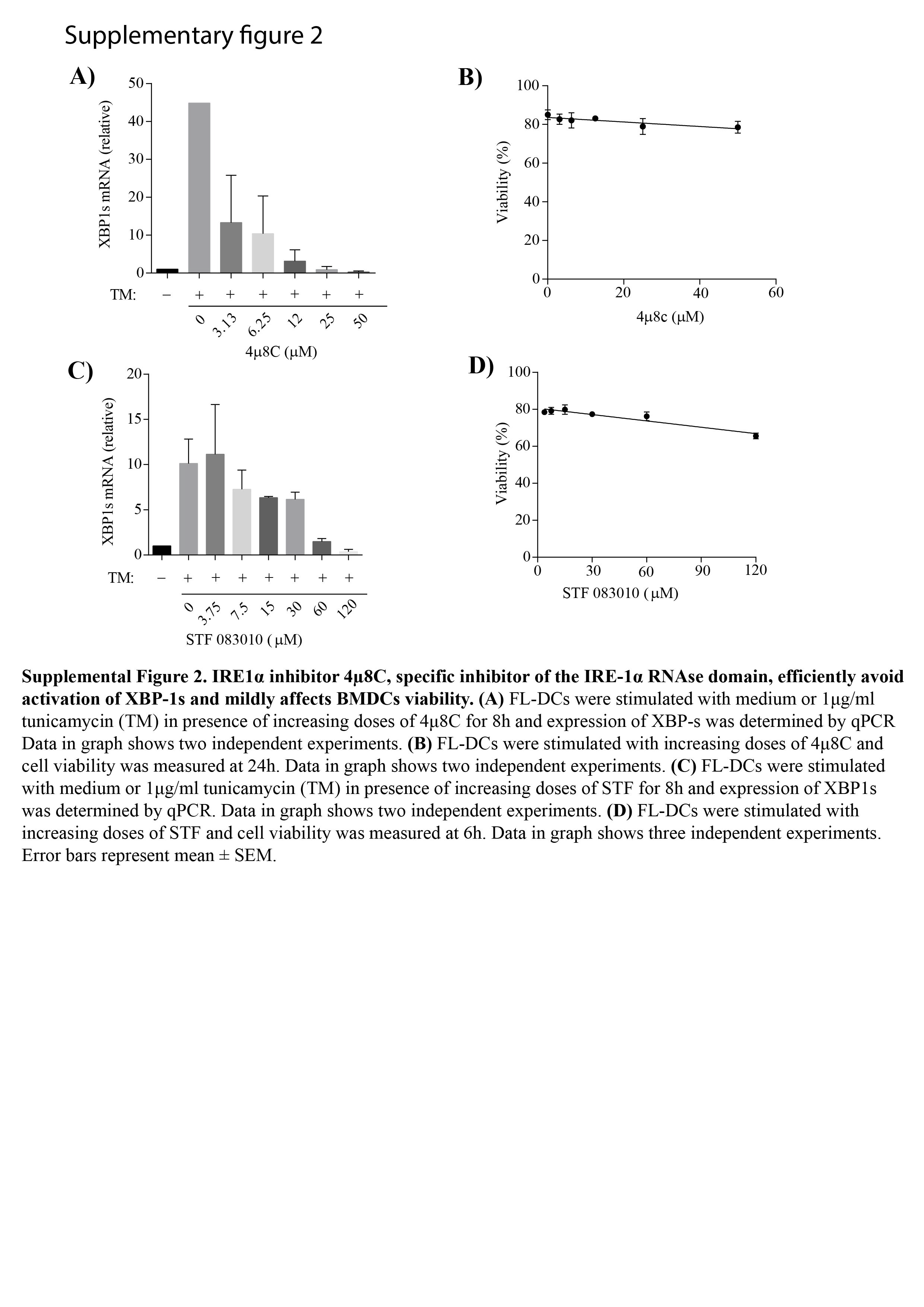

Supplement: Supplementary file 2 [file Image_2.JPEG]

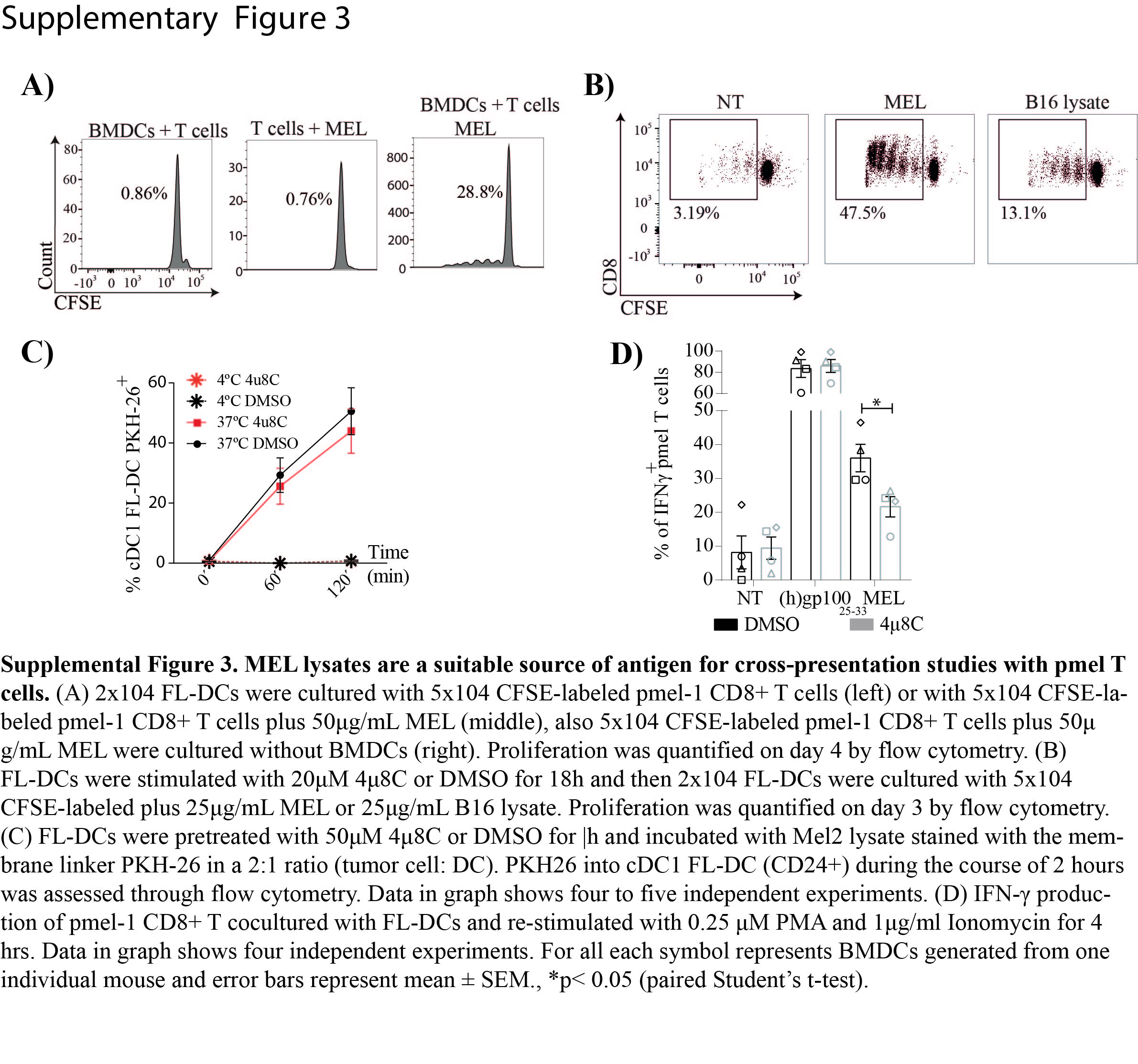

Supplement: Supplementary file 3 [file Image_3.jpg]
